# Selection of human single domain antibodies (sdAb) against thymidine kinase 1 and their incorporation into sdAb-Fc antibody constructs for potential use in cancer therapy

**DOI:** 10.1371/journal.pone.0264822

**Published:** 2022-03-03

**Authors:** Edwin J. Velazquez, Jordan D. Cress, Tyler B. Humpherys, Toni O. Mortimer, David M. Bellini, Jonathan R. Skidmore, Kathryn R. Smith, Richard A. Robison, Scott K. Weber, Kim L. O’Neill

**Affiliations:** Department of Microbiology and Molecular Biology, Brigham Young University, Provo, UT, United States of America; Duke University School of Medicine, UNITED STATES

## Abstract

Thymidine Kinase 1 (TK1) is primarily known as a cancer biomarker with good prognostic capabilities for both hematological and solid malignancies. However, recent studies targeting TK1 at protein and mRNA levels have shown that TK1 may be useful as a therapeutic target. In order to examine the use of TK1 as a therapeutic target, it is necessary to develop therapeutics specific for it. Single domain antibodies (sdAbs), represent an exciting approach for the development of immunotherapeutics due to their cost-effective production and higher tumor penetration than conventional antibodies. In this study, we isolated sdAb fragments specific to human TK1 from a human sdAb library. A total of 400 sdAbs were screened through 5 rounds of selection by monoclonal phage ELISA. The most sensitive sdAb fragments were selected as candidates for preclinical testing. The sdAb fragments showed specificity for human TK1 in phage ELISA, Western blot analysis and had an estimated limit of detection of 3.9 ng/ml for the antibody fragments 4-H-TK1_A1 and 4-H-TK1_D1. The antibody fragments were successfully expressed and used for detection of membrane associated TK1 (mTK1) through flow cytometry on cancer cells [lung (~95%), colon (~87%), breast (~53%)] and healthy human mononuclear cells (MNC). The most sensitive antibody fragments, 4-H-TK1_A1 and 4-H-TK1_D1 were fused to an engineered IgG1 Fc fragment. When added to cancer cells expressing mTK1 co-cultured with human MNCs, the anti-TK1-sdAb-IgG1_A1 and D1 were able to elicit a significant antibody-dependent cell-mediated cytotoxicity (ADCC) response against lung cancer cells compared to isotype controls (*P*<0.0267 and *P*<0.0265, respectively). To our knowledge this is the first time that the isolation and evaluation of human anti-TK1 single domain antibodies using phage display technology has been reported. The antibody fragments isolated here may represent a valuable resource for the detection and the targeting of TK1 on tumor cells.

## Introduction

Efficient DNA repair and synthesis requires a balanced supply of nucleotides and coordination of the metabolic pathways utilized for their production [1]. In order to sustain proliferation, malignant cells have significant alterations in the activity levels of several of their nucleotide synthesis enzymes [2–4]. These alterations, particularly in the pyrimidine salvage pathway, can lead to an imbalance in the cell’s nucleotide pools which could lead to error prone DNA replication and genome instability, a hallmark of cancer [5–7]. Thymidine Kinase 1 (TK1) is a pyrimidine salvage pathway enzyme that catalyzes the phosphorylation of thymidine to thymidine monophosphate [8]. In healthy cells, TK1 is only elevated during the S phase, but low or absent during other cell cycle stages [9, 10]. However, in malignant cells TK1 expression levels are upregulated and the enzyme seems to lose its normal cell cycle regulation control elements [11]. Increased levels of TK1 in both tumors and serum are associated with disease stage and, as cancer progresses, serum TK1 levels increase with disease stage [12–14]. During the last 3 decades scientific evidence has shown that TK1 levels in the serum of cancer patients can be used as a biomarker for early cancer detection [15, 16]. As TK1 levels in serum are correlated with tumor progression, patient response and cancer recurrence, TK1 has also been proposed as a suitable tumor biomarker for the continued monitoring of patients [17–21].

While the usefulness of TK1 as a tumor biomarker has been the main focus of many studies, in recent years the interest in using TK1 as a therapeutic target for multiple cancers has gradually increased [22]. In a study conducted by Malvi et al., the silencing of TK1 in lung adenocarcinoma (LUAD) cell lines inhibited the cell growth, migration and invasion capacities of LUAD cells both in vitro and in vivo [23]. Similarly, another study showed that targeting of TK1 through genetic knockdown significantly reduced cell proliferation of pancreatic ductal adenocarcinoma [24]. In addition, it has been reported, that some forms of TK1 seem to be able to associate to the cell membrane of several cancer cell types, including leukemia, breast, lung and colon tumor cells possibly through protein-protein interaction or transitory membrane localization through exosomes [25–27]. Moreover, early experimental data has shown that membrane associated TK1 (mTK1) in lung, colon and breast cancer cells could be targeted using monoclonal antibodies. However, the study was limited to some extent because the antibodies used were produced in mice and humanized antibodies are still required to better evaluate potential therapeutic use in humans [28]. This evidence together suggests that the targeting of TK1 both inside the cell and its mTK1 form could be possible approaches for the development of novel cancer therapies. Therefore, the generation of therapeutics specific for TK1 could enable us to explore the potential of TK1 as a tumor target.

Monoclonal antibodies (mAbs) are suitable candidates for the development of cancer therapies due to their high specificity and affinity for their molecule targets [29]. The majority of current therapeutic antibodies have been produced with hybridoma technology or in transgenic mice, these approaches require the use of special animals, time consuming protocols and humanization or reformatting through complicated techniques such as CDR engraftment before any therapeutic use is possible [30, 31]. Recently, phage display technology has been incorporated in the production pipeline of therapeutic antibodies by many pharmaceutical companies [32, 33]. This technology offers the possibility to explore vast human antibody libraries in a relatively short period of time compared to hybridoma technology and does not require animals and can isolate antibodies against weakly immunogenic antigens [34]. In addition, the antibody fragments can be isolated in convenient formats that facilitate further modifications for therapeutic applications [35, 36]. The use of phage display technology to obtain single domain antibody fragments (sdAbs), also called nanobodies, against TK1 is a convenient and appealing approach to isolate and develop biopharmaceuticals specific for the targeting of TK1. In this study we isolated human antibody fragments against the tumor proliferation biomarker TK1 from a sdAb library. The antibody fragments were evaluated for their capacity to bind and detect TK1 in monoclonal phage ELISAs, Western blot and flow cytometry. The antibody fragments were then incorporated into engineered IgG1 constructs and tested for their capacity to target TK1 on malignant cells and elicit an ADCC response from human MNCs against cancer cells. We hypothesize that engineered single domain antibodies (sdAb) specific for TK1 can efficiently target tumor cells expressing high levels of TK1. Thus, the use of human sdAb molecules targeting TK1 may enable us to better explore the potential of TK1 as a tumor target in proliferating malignant cells.

## Materials and methods

### Isolation of anti-TK1 sdAb fragments through phage display

A phage display library of human single domain antibodies developed by Dr. Daniel Christ at the MRC laboratory of molecular biology was used to select high affinity anti-TK1 sdAbs (Source Bioscience, Cambridge, U.K) as previously described [37]. The full repertoire of sdAbs was contained within a single human VH framework (V3-23/D47) fused to the gene III protein of the M13 filamentous phage. The sdAb library was constructed into the pR2 (MYC VSV-G tag) plasmid and had a diversity of 3x10^9^ fragments. Each sdAb fragment contained three diversified complementary regions (CDR1, CDR2 and CDR3). Ubiquitin and Galactosidase sub libraries were also included as positive controls and run before using the dAb library to test the viability of the components of the library and verify that the selection process and testing of the sdAbs would be performed correctly.

To initially amplify the full repertoire of sdAbs, one aliquot of the sdAb library, which was contained in *E*. *coli* TG1 bacteria, was grown in 500 ml of 2xTY media supplemented with 4% glucose and 100 μg/ml of ampicillin until the culture reached an OD_600_ of 0.5. An amount of 2x10^12^ KM13 helper phages were added to 500 ml of each TG1 bacteria culture, and the culture was incubated for 1 hour at 37°C without agitation. After infection, the media was replaced with 2xTY media containing 0.1% glucose, 100 μg/ml of ampicillin and 75 μg/ml of kanamycin and the library was grown for 24 hours at 25°C on a shaker at 250 rpm. The phages displaying the sdAb fragments were then purified using polyethylene glycol 6000 (PEG) solution (Millipore SIGMA, St Louis, MO, USA). The purified phages were then quantified by infecting TG1 bacteria with serial dilutions of the phage and plating the infected TG1 on TYE amp plates. Full-length human recombinant TK1 (>80% pure) produced in *E coli* (Genscript, Piscataway, NJ) was diluted in phosphate buffered saline (PBS) buffer to a 50,000 ng/ml concentration. TK1 was then immobilized on Maxisorp plates (ThermoFisher Scientific, Waltham, MA) by adding 100 ul of above TK1 solution and incubating them at 4°C overnight. The plates were then blocked with 5% milk PBS buffer (MPBS) for 45 minutes at room temperature on a shaker followed by 15 minutes at 37 ⁰ C. The plates were then washed 3 times with PBS. Approximately 5x10^10^ phages displaying the full repertoire of sdAbs, were applied to each well that had been coated with TK1. To increase the surface area available for capture of the whole repertoire of the sdAb library, each phage dilution was added to four wells. Phages were allowed to bind for 1 hour at room temperature with moderate shaking. After incubation, the wells were washed 15 times with PBS-T buffer (0.1% Tween) and 2 times with PBS buffer. The phage-sdAbs that remained attached were then eluted by adding 100 μl of a 0.1 mg/ml trypsin-TBSC buffer solution (Millipore SIGMA, St Louis, MO, USA) and incubation for 1 hour at room temperature with moderate shaking. The eluted phages were then recovered and used to infect a 30 ml TG1 bacteria culture 0.5 OD_600_. The infected culture was then incubated for 1 hour at 37 ⁰C without shaking. After infection, the TG1 bacteria were harvested and resuspended in 1 ml of 2xTY media. The cells were then plated on TYE, 4% glucose plates with ampicillin (100 μg/ml). The next day, colonies were scraped off and grown in 500 ml of 2xTY until the cultures reached 0.5 OD_600_. The cultures were then infected with 1x10^12^ KM13 phages and grown at 25 ⁰C for 24 hours on a shaker at 250 rpm. After incubating for 24 hours, the phage-antibody sub library was purified using PEG 6000 and the phage titer was determined for both the eluted phages and the purified phages. This process of selection was repeated 5 times. To eliminate antibodies that could possibly bind to the 6xHis-tag in TK1, the last two rounds of selection were done using TK1 produced in HEK 293expi cells without a 6xHis-tag (Origene, Rockville, Maryland, USA).

### Polyclonal and monoclonal phage ELISAs

To monitor the increase in the overall number of TK1 binders between rounds of selection the isolated phages from each round of selection were screened using a polyclonal phage ELISA. For the polyclonal phage ELISA 96-well Costar plates (Corning, NY, USA) were coated with 100 μl of serial dilutions of TK1 (50,000 ng/ml-400 ng/ml) in duplicates. Uncoated wells (without antigen) were included as blanks in each ELISA. After the wells were coated, the plates were incubated overnight at 4°C with gentle agitation. After incubating, the plates were washed 3 times with PBS buffer and blocked with 240 μl of MPBS for 45 minutes at room temperature followed by 15 minutes at 37°C. Wells were washed 3 times with PBS and purified phages diluted in MPBS (1:1 ratio) were added into each well. The plates were incubated for 1 hour at room temperature with gentle agitation. After incubating the plates were washed with PBS-T five times and 100 μl of Horse Radish Peroxidase (HRP) conjugated anti-M13 antibody solution (1:2,000 dilution in MPBS) was added into each well. Following addition of the HRP conjugated antibody, 100 μl of Tetramethyl Benzidine (TMB) substrate (ThermoFisher scientific, Waltham, MA, USA) was added into each well and the color was allowed to develop for 30 minutes. The reaction was stopped with 50 μl of 1M sulfuric acid, and the absorbance values were measured using a Synergy HT Microplate Reader (Bio-Tek Winooski, VT) at 450 nm and 650 nm.

Eighty individual clones were tested using monoclonal phage ELISA following every round of selection (biopans). After each biopan and before scraping off the colonies, eighty clones were picked and grown overnight at 37°C in 200 μl of 2xTY media supplemented with 4% glucose and ampicillin (100 μl/ml). Culture dilutions (1:40) from the overnight cultures were made by diluting 5 μl of the overnight culture in 200 μl of 2xTY 4% glucose and ampicillin and grown at 37°C for three hours. After reaching a 0.5 OD_600_, 50 μl of 2xTY containing 4 x 10^8^ KM13 helper phages were added into each well to produce phage-sdAb fragments. The infected cultures were incubated at 37°C for one hour without shaking and the media was changed with 200 μl of 2xTY with 0.1% glucose, ampicillin (100 μg/ml), and kanamycin (75 μg/ml). Cultures were grown at 25°C for 24 hours at 250 rpm. After 24 hours the plates were centrifuged at 3200 xg for 10 minutes and the supernatants were recovered from each well. Each supernatant was then mixed with MPBS in 1:1 ratio. To test each clone for its capacity to bind to TK1, 96 well plates were coated with 100 μl of a 10,000 ng/ml solution of TK1 per well as previously described. The same protocol as described for the polyclonal phage ELISA was used for the monoclonal phage ELISA.

### Detection of phage-sdAbs by dot blot

Dot blot was used to confirm the expression of sdAb fragments in TG1 supernatants. After recovering supernatants containing phage-sdAb fragments, 2–3 μl of each supernatant containing the phage-sdAbs was immobilized on a nitrocellulose membrane (BIO-RAD, Hercules, CA). The membrane was then blocked with 5% MPBS for 1 hour at room temperature on a shaker. The membrane was then incubated with a 1:20,000 anti-VSV-G-HRP antibody solution at 4°C overnight with moderate shaking. The next day, the membrane was washed 3 times with 240 μl of PBS-T for 5 minutes. After the membranes were washed, 2 ml of enhanced chemiluminescence substrate (Advansta Corporation, San Jose, CA) was added so that the membrane was completely covered. The membranes were incubated for 2 minutes, and the excess of reagent was poured off. Membranes were covered in plastic wrap and light sensitive films were placed on the membranes for different exposure times and measured using an imaging developer.

### Sensitivity of TK1 specific antibody fragments

The sensitivities of the TK1 sdAbs were determined using dose-response curves and monoclonal phage ELISA. Briefly, Costar 96-well plates (Corning) were coated with serial dilutions of TK1 in duplicate overnight at 4⁰C with gentle agitation. The dilutions ranged from 23,600 ng/ml-23 ng/ml for E-TK1 and 500 ng/ml-3.9 ng/ml for H-TK1. The next day, plates were blocked with 240 μl of MPBS/well for 45 minutes at room temperature and 15 minutes at 37°C. After blocking, the wells were washed three times with PBS and 100 μl of a 1:1 dilution of phage supernatant in MPBS was added into each well. Subsequent steps in the ELISA were carried out as previously described. The curves were then analyzed using a four-point parameter logistic curve and the limit of detection of each sdAb fragment was determined. A signal that was at least three standard deviations away from the signal of the blank was used as the limit of detection, as previously described [38]. The sensitivities of the clones were then compared.

### Validation with a TK1 siRNA and non-specific binding controls

In order to confirm specific binding to TK1, individual clones were screened against lysate from a siRNA TK1 knockdown cell line and compared to wild type cell lysate. The TK1 knock down cell lysate was prepared as previously described [28]. TK1 forms produced in bacterial, yeast, and mammalian expression systems were used as positive controls and uncoated wells were used as negative controls. The monoclonal phage ELISA was performed as described above.

### Sequencing analysis of sdAb fragments

Plasmids were isolated from the clones that showed the highest affinity for TK1 using the PureYield Plasmid Miniprep system (Promega). Samples were prepared for sequencing using the primer (5’ CCCTCATAGTTAGCGTAACGA 3’) and the universal M13 reverse primer (5’ CAGGAAACAGCTATGAC 3’). Sequencing data was analyzed using Genious prime software [39].

### Anti-TK1-sdAbs protein modeling and docking analysis of sdAb-TK1complexes

The structures of the anti-TK1-sdAb fragments were analyzed using the GalaxyWEB TBM web server. The most stable structures of each anti-TK1 sdAb fragment were analyzed using the Visual Molecular Dynamics (VMD) software developed by the computer science and biophysics department of the University of Illinois [40]. The CDR regions were mapped by analyzing the deduced amino acid sequences of each anti-TK1-sdAb fragment in the IgG Blast tool from NCBI. The sequences of the anti-TK1-sdAb fragments were aligned using Genious prime software. In silico analysis of the interaction between the anti-TK1-sdAb fragments and the crystal structure of TK1 was performed using the high ambiguity driven protein-protein docking (HADDOCK) web server [41]. Visualization of the anti-TK1-sdAb-TK1 complexes was also performed with VDM software.

### PCR amplification and cloning into pET-scFv-T

Sequences of the sdAb fragments were amplified from the phagemid plasmids corresponding to the isolated positive clones in phage ELISA. The sequences were amplified using primers containing the NcoI restriction site (5’ GAACATATGATGAAAAAATTATTA 3’) and the NotI restriction site (5’ GAAGGATCCTGCGGCCCCCTTTC 3’). PCR products were run in a 1% agarose gel and desired sequences were extracted using the Zymoclean DNA Gel Recovery Kit (Zymo research, Irvine, CA, USA). Following gel recovery, sdAb sequences were digested using NcoI and NotI restriction enzymes (New England Biolabs, Ipswich, MA, USA). Digested sequences were then ligated into the pET-scFv-T backbone (Addgene, Watertown, MA). Ligation was carried out using the Quick Ligation kit (New England Biolabs, MA). The ligated plasmids were then cloned into Dhα5 competent cells. Transformed colonies were grown for 16–20 hours and plasmids were isolated and analyzed by restriction enzyme analysis to verify the presence of the insert. Positive clones were then sequenced.

### Expression of antibody fragments in Rosseta 2(DE3) pLysS E. coli cells and His-tag purification

The pET-TK1-sdAb-6xHis constructs were cloned into Rosseta blue (DE3) pLysS *E*. *coli* cells (Millipore SIGMA, St Louis, MO). Individual colonies were selected and grown in a culture overnight at 37°C. The overnight culture was then scaled up and grown until the OD_600_ reached 0.6. Expression of the sdAb fragments was induced by addition of 0.4 mM Isopropyl β-d-1-thiogalactopyranoside (IPTG). After IPTG induction, the cultures were grown for 24 hours at 28°C. The Rosseta blue(DE3) pLysS *E*. *coli* cells were pelleted and the supernatant was saved for later purification. The cells were subjected to osmotic shock by resuspending cells in TES buffer (20 mM Tris-HCl pH 7.6, 5 mM EDTA, and 20% sucrose). After one hour incubation on ice, the sample was centrifuged at 14,000 xg for 20 minutes. The pellets were resuspended in ddH_2_O and incubated on ice for 30 minutes. Centrifugation was repeated and the supernatant was preserved. The cell pellet was lysed using 10x bug buster reagent (Millipore SIGMA, St Louis, MO) diluted in 20 mM Tris–HCl (pH 7.8) buffer containing 15 mM NaCl, 5 mM MgCl_2_, DNase (25 U/ml) and protease inhibitors. Cells were lysed for 20 minutes on shaker with moderate agitation. The lysed cells were spun down at 16,000 g for 20 minutes and the supernantant was recovered. Supernatants were mixed with equilibrated Ni-NTA agarose beads (Qiagen, Hilden, Germany) for 3 hours at 4°C. After incubating the NI-NTA beads were washed twice with cell lysis buffer and placed into 5 ml polyproylene columns. The Ni-NTA beads were then washed with 50 ml of wash buffer and then the His-tagged proteins were eluted with elution buffer in 0.3 ml fractions. The fractions were analyzed by SDS-PAGE and Western blot to detect the purified anti-TK1 sdAb fragments and estimate their purity.

### sdAb ELISA

TK1 sdAbs expressed in a pET system and purified were tested again in ELISA to confirm that their binding properties to TK1 were kept when expressed without being fused to the PIII coat protein of the phages. The ELISA was performed as previously described for phage ELISA, except the blocking solution was replaced with 5% BSA. Because these antibodies were expressed with a VSV-G-tag this time the detection antibody was an anti-His-HRP (Biolegend, San Diego, CA) or anti-VSV-G-HRP (Bethyl, Montgomery, TX) rather than an anti-M13-HRP antibody.

### Western blot with purified anti TK1-sdAb fragments

The purified fragments were tested for their capacity to bind to purified TK1 and TK1 in cell lysate using Western blotting. Recombinant human TK1 produced in bacteria and Expi293F cells were used together with cell lysate from A549 lung cancer cells, including a siRNA TK1 knockdown cell lysate. Briefly, 0.5 μg of TK1 or 20 μg of cell lysate were mixed with 6x Laemmli buffer (Millipore SIGMA, St Louis, MO). The protein samples were then heated at 100°C for 5 minutes and loaded into a 12% SDS-PAGE electrophoresis. The proteins from the gel were then transferred to nitrocellulose membranes (Bio-Rad, Hercules, CA, USA). After blocking with 5% milk in MPBS buffer for 1 hour at room temperature the blocking solution was poured off and anti-TK1 sdAb fragment solution (1 μg/ml-2 μg/ml) was added. Membranes were incubated at 4°C overnight. After overnight incubation membranes were washed 3 times with PBS-T buffer. The bound proteins were detected using a 1:20,000 solution of an anti-VSV-G-HRP and anti-His-HRP antibody (Bethyl, Montgomery, TX, USA). The proteins were then detected through the peroxidase reaction using enhanced chemiluminescence (ECL) (Advansta Corporation, San Jose, CA). Films were exposed for differing amounts of time depending on the antibody being tested, times ranged from 30 seconds to 5 minutes. The films were scanned, and the images were analyzed using the software ImageJ from NIH.

### Cell lines and isolation of human MNCs

The NCI-H460 (ATCCHTB-177^TM^), A549 (ATCC^®^ CCL-185), HCC1806 (ATCC^®^ CRL-2335^TM^) and HT-29 (ATCC^®^ HTB-38^TM^) cell lines were obtained from the American Type Culture Collection (ATCC, Manassas, VA, USA) and maintained according to ATCC recommendations. A549, NCI-H460, HCC1806 and HT-29 were cultured in RPMI-1640 media (ThermoFisher scientific, Waltham, MA) supplemented with 10% fetal bovine serum (FBS) and 2mM L-Glutamine. All cell lines were grown in an incubator at 37°C and 5% CO_2_. All cell lines used were tested for TK1 surface expression with flow cytometry with the commercial antibody ab91651 (Abcam, Cambridge, UK) to confirm the presence of TK1 on the cell membrane. MNCs were isolated using lymphocyte separation media (Corning, NY) following the instructions from the product’s manual, red blood cells were depleted with red blood cell lysis buffer (Biolegend, San Diego, CA) and the MNCs were resuspended in LGM-3 (Lonza, Basel, Switzerland). Isolation of human MNCs and serum from blood was done under approval of the Brigham Young University Institutional Review Board number 1734.

### Flow cytometry

The purified phage-sdAb fragments, anti-TK1 sdAb fragments, and anti-TK1-sdAb-IgG1 fusions were all tested for their capacity to detect mTK1 on the cancer cell lines NCI-H460, HCC1806, HT-29, and normal MNCs. For this analysis 1x10^6^ cells per sample were analyzed. Cells were washed twice with PBS and resuspended in 200 μl of cell staining buffer. After resuspension, the cells were stained using PEG purified phage-TK1-sdAb fragments (100 μl), purified sdAb fragments (5–10 μg), or purified sdAb-IgG1 antibodies for 40 minutes. The cells were then washed 3 times with 200 μl of cell staining buffer and stained for 30 minutes with anti-his-APC or anti-VSV-G-FITC antibodies or, in the case of purified phage-sdAb fragments, anti-M13-FITC secondary antibody. To detect binding of sdAb-IgG1 antibody fusions anti-Human IgG-FITC antibody (Abcam, Cambridge, UK) was used. After incubation with secondary antibody, the cells were washed 3 times with 200 μl of cell staining buffer. Before analysis samples were stained with 10 μg/ml PI solution and the samples were analyzed in a Cytoflex flow cytometer machine (Beckman Coulter, Brea, CA). The FCS files were analyzed using the FlowJo software (FlowJo, Inc., Ashland, OR).

### Incorporation of TK1-sdAb fragments into pFUSE-IgG1 constructs and antibody expression in CHO cells

The DNA sequences of the best two anti-TK1 sdAbs were cloned between the NcoI and EcoRI restriction sites of the pFUSE-hIgG1e5-Fc2 vector (InvivoGen, San Diego, CA). This vector is designed for the production of human recombinant antibodies in mammalian cells and contains a human IgG1 heavy chain mutated at the S239D/A330L/I332E sites which confers an increased binding to FcγIIIa receptors in macrophages (MO) and natural killer cells (NK). Thus, the recombinant antibodies fused to this engineered IgG1, can elicit an enhanced antibody-dependent cell-mediated cytotoxicity (ADCC). The primers used for the amplification of the sdAb fragments for this construct were primer Fw- 5’ GAAGAATTCGATGGCCGAGGTGCAG 3’ and primer Rv- 5’ GGCCCATGG CGCTCGAGACGGTGAC 3’. The two TK1-scFv-hIgG1 DNA constructs were introduced into CHO.K1 cells (ATCC, Manassas, VA, USA) by lipofection using the lipofectamine LTX reagent (ThermoFisher scientific, Waltham, MA). After 48 hours the media was changed and Zeocin selection antibiotic (InvivoGen, San Diego, CA, USA) was added at a concentration of 100 μg/ml. After 10 days in selection the cells were expanded. The media was then replaced with ProCHO^TM^ AT (Lonza, Basilea, Switzerland) and the cells were incubated for 48–96 hours or until cell viability was about 50%. The media was collected and cleared from cells by centrifuging at 200 xg. The anti-TK1-scFv-hIgG1 antibodies where then purified from cleared media using protein A purification columns (ThermoFisher scientific, Waltham, MA). Characterization of the purified recombinant antibodies was carried out with Western blot and flow cytometry as described above.

### In vitro testing of anti-TK1-sdAb-IgG1 antibodies through ADCC

The capacity of the TK1-sdAb-IgG1 antibodies to target mTK1 on cancer cells and elicit an ADCC response was evaluated *in vitro*. For this experiment NCI-H460 cells, which expressed high levels of mTK1, were engineered to express cytosolic GFP. The cells were then co-cultured with human MNCs and anti TK1-sdAb-IgG1 antibodies were added in various concentrations. Cell death was then measured using a real time cell imaging system. The experiments were conducted as follows. One day before treating the cells with antibodies and controls 5000 NCI-H460 GFP+ cells were seeded per well in a 96-well tissue culture plate (MIDSCI, St. Louis, MO), placed inside an ImageXpress^®^ Pico system. The GFP+ cells were counted every hour for the next 8–12 hours. After initial growth human MNCs were added at two different ratios, 5:1 and 10:1. The cells were co-cultured using LGM-3 media to sustain MNCs and antibodies were added at various concentrations (20, 10, 5, 2.5 μg per well). An optimized 5:1 effector:target ratio and a concentration of 10 μg of antibody/ml were used for experiments. The cells were monitored for 72–96 hours under environmentally controlled conditions. The number of cells from each treatment including controls were analyzed and compared over time. Each treatment in the assay was run in duplicate wells and the experiment was repeated twice.

### Statistical analysis

Statistical analyses were performed using the GraphPad Prism software (GraphPad, San Diego, CA). ELISA data from dilution curves was log-transformed and analyzed with a 4-parameter non-linear regression analysis with a 95% confidence interval (CI). To compare the different treatments of the ADCC experiments, the data was normalized in reference to the moment MNCs and antibodies were added and analyzed using a two-way ANOVA with repeated measure analysis. Analysis of multiple comparisons was performed comparing the mean of each treatment with every other treatment mean in each time point and over the total course of time. One-way ANOVA were performed to compare normalized GFP+ cell counts at specific time points.

## Results

### Antigen validation

**Fig 1:**
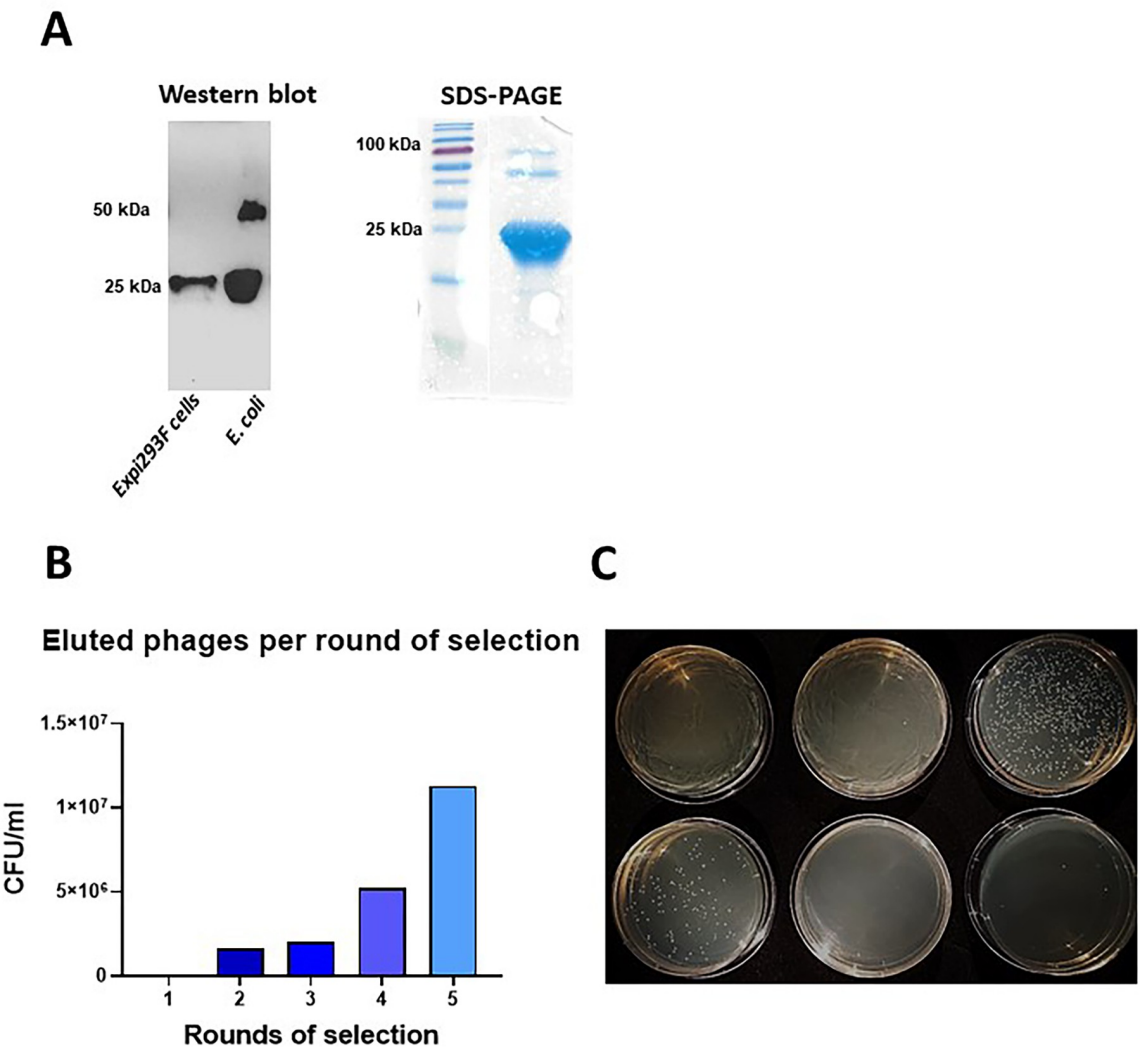
Antigen validation and quantification of eluted phages thorough rounds of selection A) Antigen validation, the purity and integrity of human recombinant TK1s produced in *E*. *coli* cells and Expi293F cells were assessed in SDS-PAGE and validated using Western blot with the anti-TK1 antibody ab91651. B) Enrichment of TK1 binders through 5 rounds of selection. The number of binders was estimated based on titrations of eluted phages used to infect TG1 bacteria. C) A representative image of a viral titration to determine the number of eluted phages. TG1 bacteria were infected with serial dilutions of the eluted phages after each biopan. The infected TG1 bacteria were then plated on TYE amp plates.

All of the antigens used for the selection of the sdAbs were validated before the biopanning process. Recombinant human TK1 was produced in *E*. *coli* (E-TK1) and was used during early rounds of selection at high concentrations while TK1 produced in human Expi293F cells (H-TK1) was used for the last two rounds of selection at lower concentrations. This is because H-TK1 produced in human cells is properly folded. Both antigens were validated using Western blot with the KO validated anti-TK1 antibody ab91651 (Abcam, Cambridge, UK). Both E-TK1 and H-TK1 purified fractions were positive for TK1 and showed bands respective to the monomer and dimer of TK1. The purity of the antigens was shown to be higher than 80% based on the SDS page and Coomassie blue staining (Fig 1A, right).

### Isolation and validation of anti-TK1 sdAb fragments

The isolation of anti-TK1-sdAbs was monitored after each biopan by determining the phage titer from the eluted phages. As expected, the number of eluted phages per ml increased exponentially through the 5 rounds of selection (Table 1, Fig 1B). This trend could be observed by infecting TG1 bacteria with the eluted phages, plating the bacteria in selective agar plates and then determining the number of CFU/ml (Fig 1C). After 3 consecutive rounds of selection with recombinant human TK1 produced in *E*. *coli* the enrichment factor of TK1 binders went from 1 to 48.9. (Table 1). Two more rounds of selection were performed using recombinant H-TK1 to eliminate non-specific sdAb fragments that could possibly bind to the His-tag present in E-TK1 and also to obtain fragments that could bind to properly folded human TK1. We observed that the enrichment factor increased 3-fold after 1 round of selection with H-TK1 and then doubled in the subsequent round of selection using H-TK1.

**Table 1:** Enrichment of anti-TK1-sdAb phages through 5 rounds of selection.

Round of selection	Input phages	eluted phages	Ration (eluted/input)	Enrichment factor
1	5.00E+12	4.12E+04	8.24E-09	1.00
2	5.00E+12	1.60E+06	3.20E-07	38.83
3	5.00E+12	2.01E+06	4.03E-07	48.91
4	5.00E+12	5.20E+06	1.04E-06	126.21
5	5.00E+12	1.13E+07	2.27E-06	275.49

**Fig 2:**
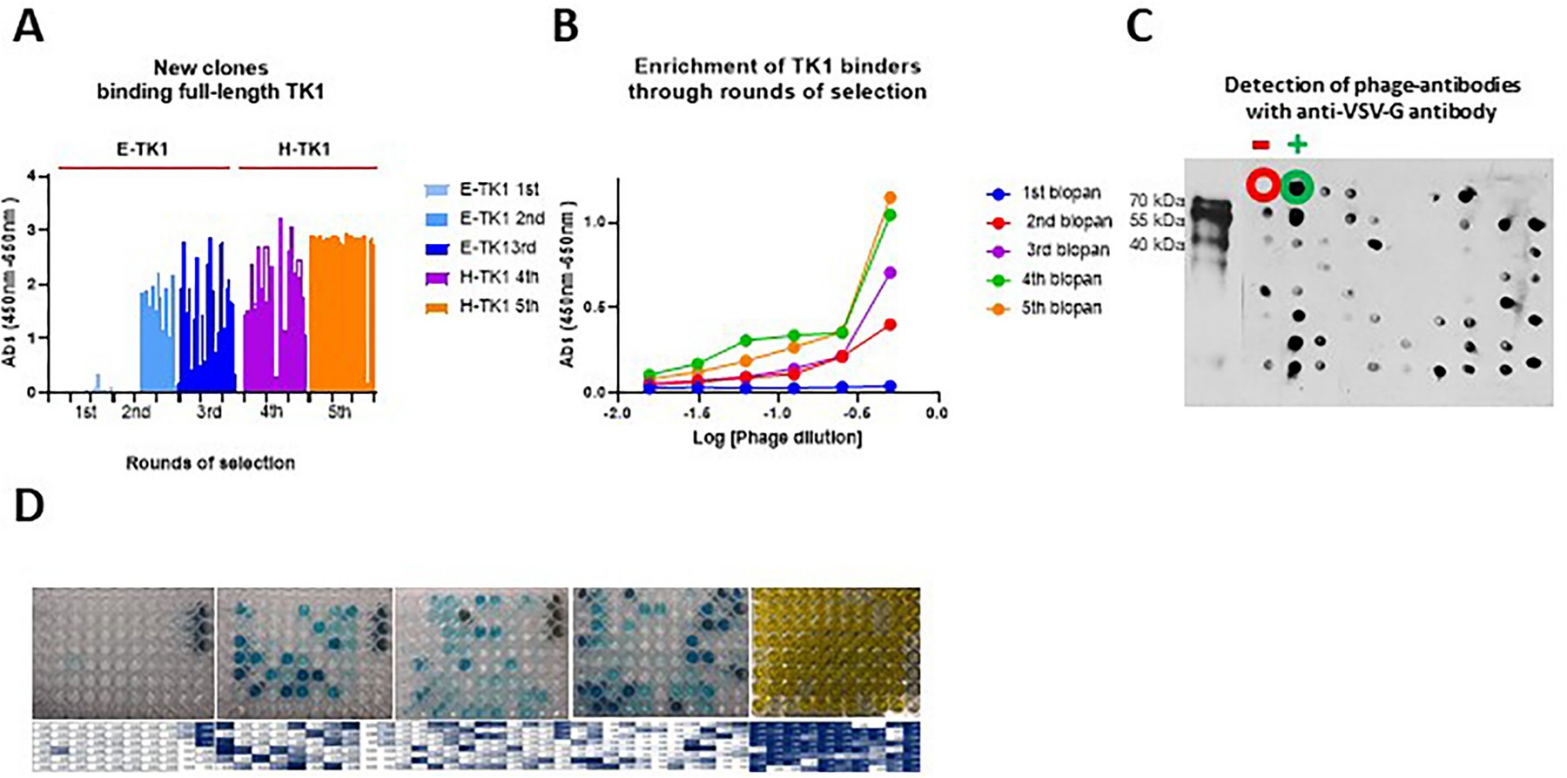
Selection and expression of anti-TK1-sdAb phages. A) Analysis of 80 clones using monoclonal phage ELISA was performed after each round of selection. B) Polyclonal phage ELISA using the total purified phages per round of selection. In both A and B the overall signal and number of positive clones increased after each round of selection. C) Detection of anti-TK1-sdAb phages using dot blot with an anti-VSV-G-HRP antibody. Also, verification of packaging of the library from PEG purified phages after initial amplification of the sdAb library. D) Representative image of monoclonal phage ELISAs during the rounds of selection.

After each biopan, 80 individual clones were screened through monoclonal phage ELISA. The purified phages were also monitored after each round using polyclonal phage ELISA. As expected, an exponential increase in the number of positive clones after each biopan was observed. About 50% of the clones produced a positive signal by the 4^th^ biopan and about 90% of the clones were positive in the 5^th^ biopan (Fig 2A). A similar trend was observed in polyclonal phage ELISA where the signal produced by the total purified phages after each round of selection also significantly increased after the first biopan and kept increasing almost 2-fold between each biopan from the 2^nd^ to the 4^th^ biopans (Fig 2B). Although the number of positive clones in monoclonal phage ELISA showed a significant increase from the 4 to the 5^th^ biopan, no significant increase in the overall signal was produced in polyclonal phage ELISA. This was consistent with what was observed in the monoclonal phage ELISAs where we see significant increases in the number of positive clones from the 1^st^ through the 4^th^ biopans.

In addition to monoclonal and polyclonal phage ELISAs, we screened purified phages and individual supernatants containing phages with Western blot and dot blot. Since each antibody fragment displayed in the M13 phages had Myc and VSV-G tags, we detected the production of phage-antibodies using anti-VSV-G-HRP conjugated antibody. Western blot analysis of the purified phages revealed the presence of bands corresponding to phage-sdAb fusions. Dot blot analysis of individual clones showed the successful production of individual phage-sdAbs (Fig 2C).

**Fig 3:**
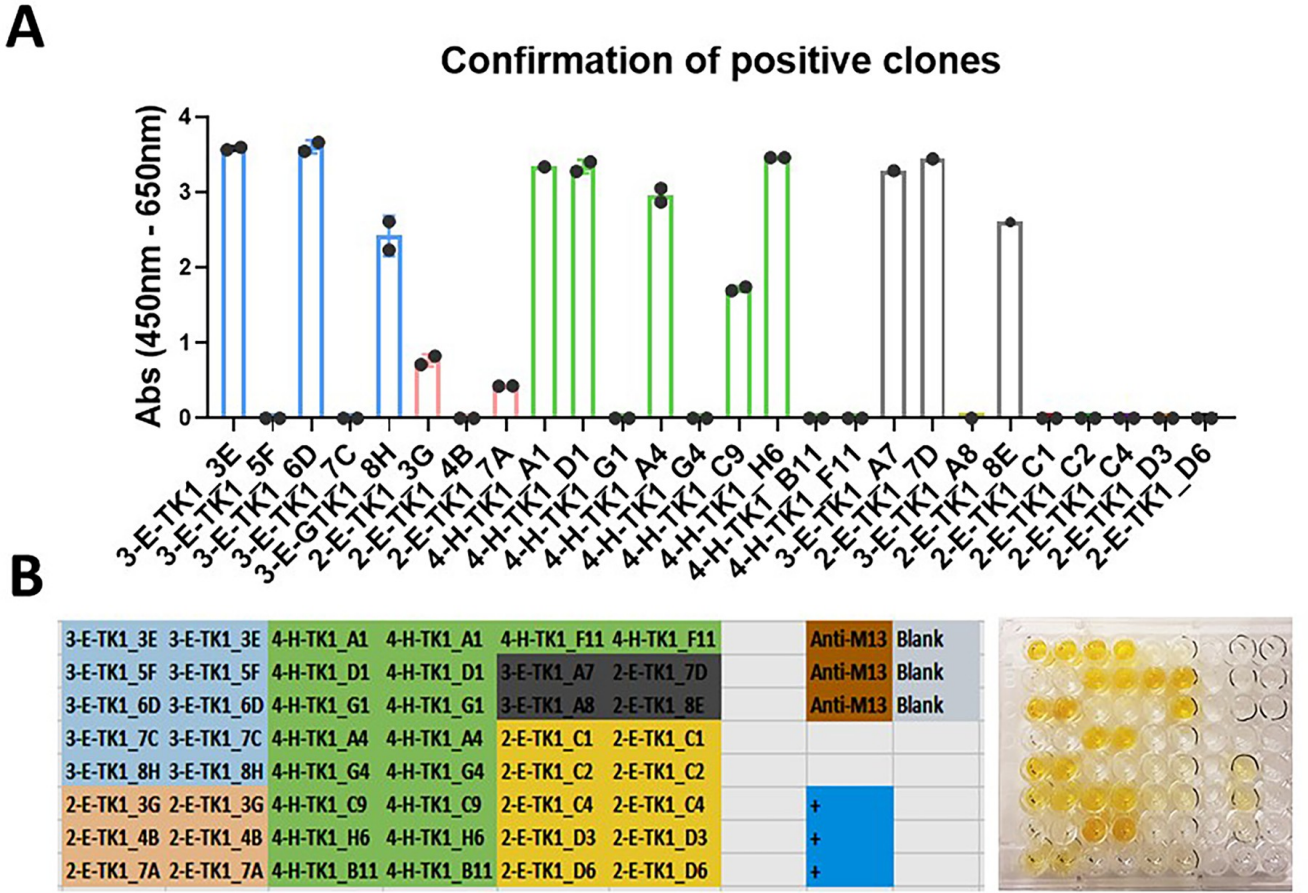
Confirmation of positive clones. To verify the positive clones found during the initial screening, their capacity to reproduce a positive signal was tested. The strongest positive clones were selected through the different rounds of selection. Bacteria corresponding to each of these clones were streaked for a second time on TYE amp plates. New cultures were grown from a single colony and infected with KM13 helper phage to induce the production of their respective anti-TK1 phage-sdAbs. A) Positive anti-TK1 phage-sdAbs tested in monoclonal phage ELISA. The capacity to bind TK1 and the stability of each clone was confirmed. B) Representative image showing the color development generated by the positive clones and plate layout indicating the position of each clone that was tested.

After the initial screens using monoclonal phage ELISA, 26 clones were chosen for their ability to produce high signals. These included clones from the 2^nd^, 3^rd^ and 4^th^ rounds of selection. Clones from the 5^th^ biopan were excluded due to a decrease in the diversity of the sdAb sequences. The clones were re-tested to confirm their capacity to bind TK1 and reproduce a positive signal. From the 26 selected clones, 14 clones were able to reproduce a positive signal after the initial screen (Fig 3).

**Fig 4:**
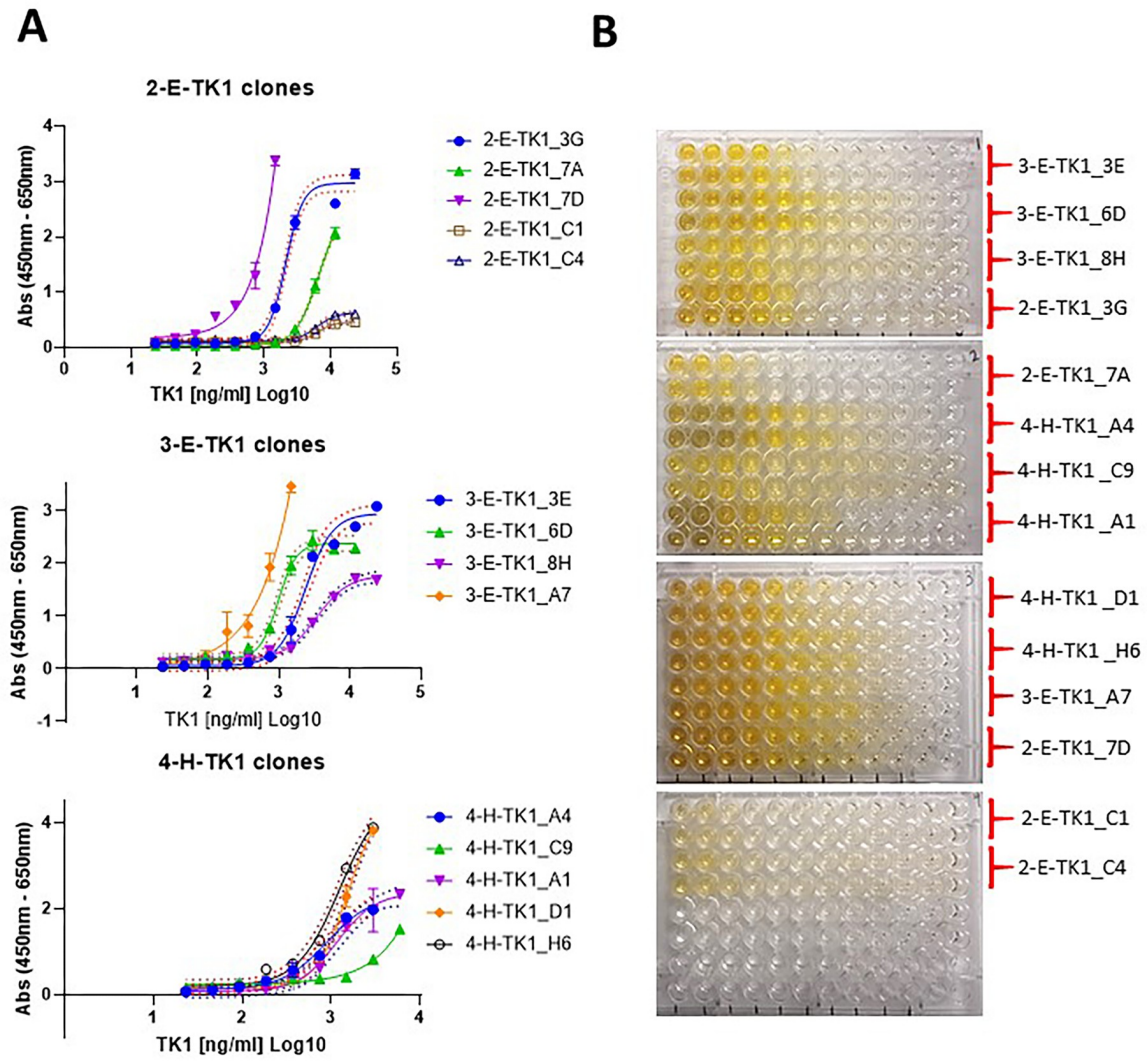
Testing of the anti-TK1 sdAbs with dose response curves. A) Dose response curves corresponding to anti-TK1 sdAb fragments obtained through various rounds of selection. The data was Log transformed and the curves were analyzed using a 4-parameter non-linear regression. B) A representative image of the colorimetric reaction showing that the signal of each anti-TK1 sdAb fragment is proportional to the concentration of TK1 protein. Negligible or no significant signals were produced in the blanks.

After confirming their binding to TK1, the positive clones were evaluated using dose calibration curves to determine if they bound to TK1 quantitatively and to determine their sensitivity. The 14 clones showed all sigmoidal curves according to our non-linear 4-point logistic analysis. The goodness of fit test showed R squares ranging between 0.9964–0.9772. The curves behaved according to receptor-ligand interactions models, showing that the binding of the anti-TK1-sdAbs were proportional to the concentration of TK1 in each well (Fig 4A). This could also be visually appreciated in the colorimetric reactions in the dilution curves for each clone (Fig 4B). There was no significant background signal produced by the clones in the blanks.

**Fig 5:**
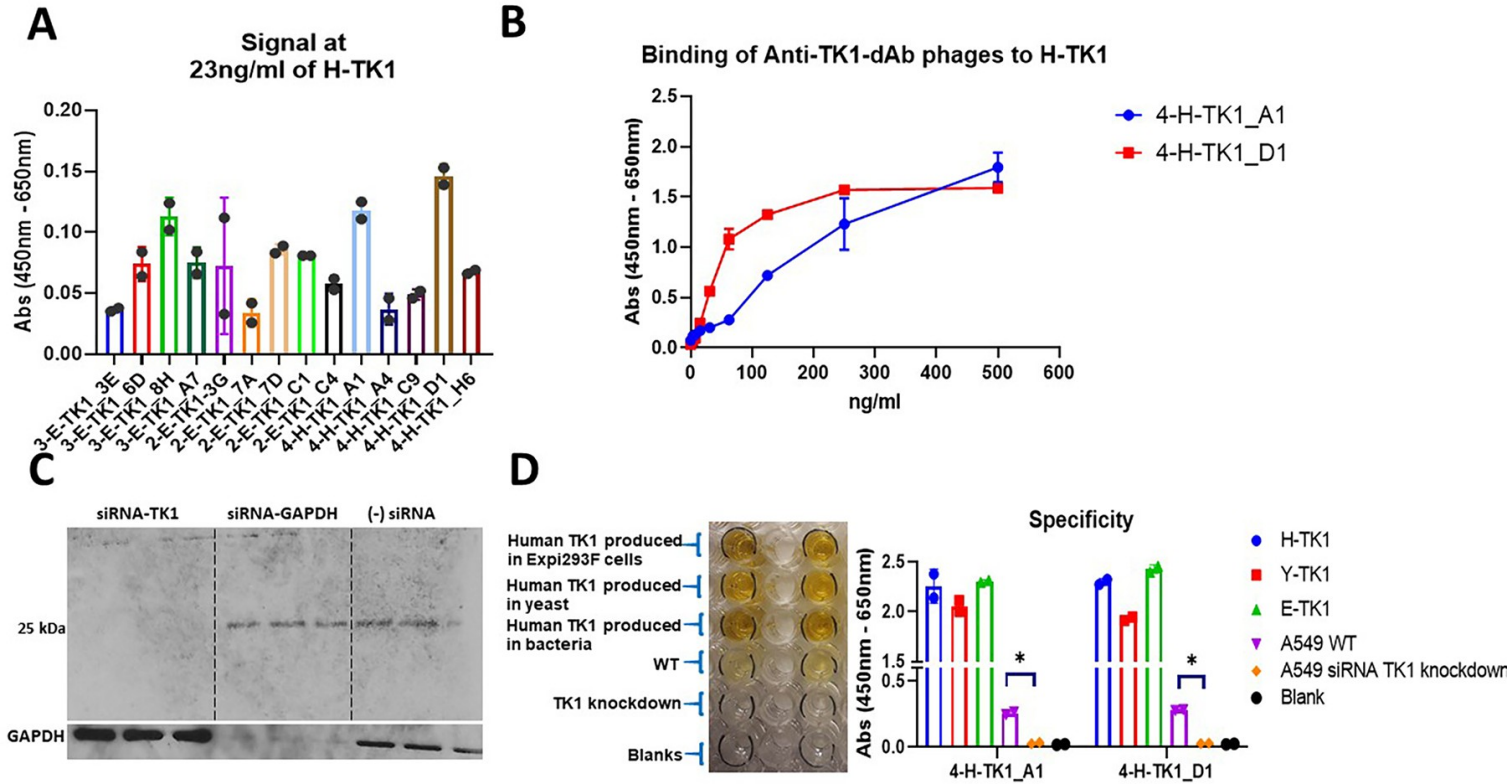
Sensitivity and specificity of the anti-TK1-sdAb fragments. A) The 14 anti-TK1 sdAb fragments were tested against a minimal fixed concentration of 23 ng/ml of human TK1 produced in human cells (H-TK1). The sdAbs 4-H-TK1_A1 and 4-H-TK1_D1 produced the highest signals. B) Dilution curves with H-TK1 and the best clones. Concentrations ranged from 500 ng/ml to 3.9 ng/ml, the fragments kept their binding properties after being expressed as sdAb fragments C) siRNA TK1 knock down validation. TK1 was knocked down in A549 cells. The knockdown was validated using the commercial anti-TK1 antibody ab91651. D) Validation of the best 2 anti-TK1-sdAbs. The binding capacity of the sdAb fragments was tested against 3 different sources of human recombinant TK1, cell lysate from the cancer cell line A549 and cell lysate from A549 TK1 knockdown. It can be seen that both fragments bind to all recombinant TK1 proteins. A significant difference can be seen in the signal coming from the normal cell lysate in comparison with the TK1 knockdown cell lysate.

The most sensitive clones were 4-H-TK1_A1 and 4-H-TK1_D1. Using the phage supernatant from these clones we were able to detect TK1 protein levels as low as 23 ng/ml in monoclonal phage ELISA (Fig 5A). Further sequencing of the clones revealed that the sequences of clones 4-H-TK1_A1 and 4-H-TK1_D1 were different.

Anti-TK1-sdAbs phages 4-H-TK1_A1 and 4-H-TK1_D1 demonstrated the capacity to bind H-TK1 in phage ELISA. The signal was proportional to H-TK1 concentration (Fig 5B). Validation of the clones 4-H-TK1_A1 and 4-H-TK1_D1 using a siRNA TK1 knockdown and different sources of recombinant human TK1 revealed that the clones were able to bind to E-TK1(produced in *E*. *coli*), H-TK1(produced in Expi293F cells) and TK1 produced in a yeast system. Moreover, the signal with cell lysate from A549 cells was significantly stronger (~10-fold) to the signal coming from the cell lysate of A549 TK1 knockdown for both 4-H-TK1_A1 (*P*<0.0296) and 4-H-TK1_D1 (*P*<0.0129). A TK1 knock down was produced as previously described for this experiment (Fig 5C and 5D) [28].

### Amplification of sdAB fragments, sequencing and cloning into pET-scFv-T vector

**Fig 6:**
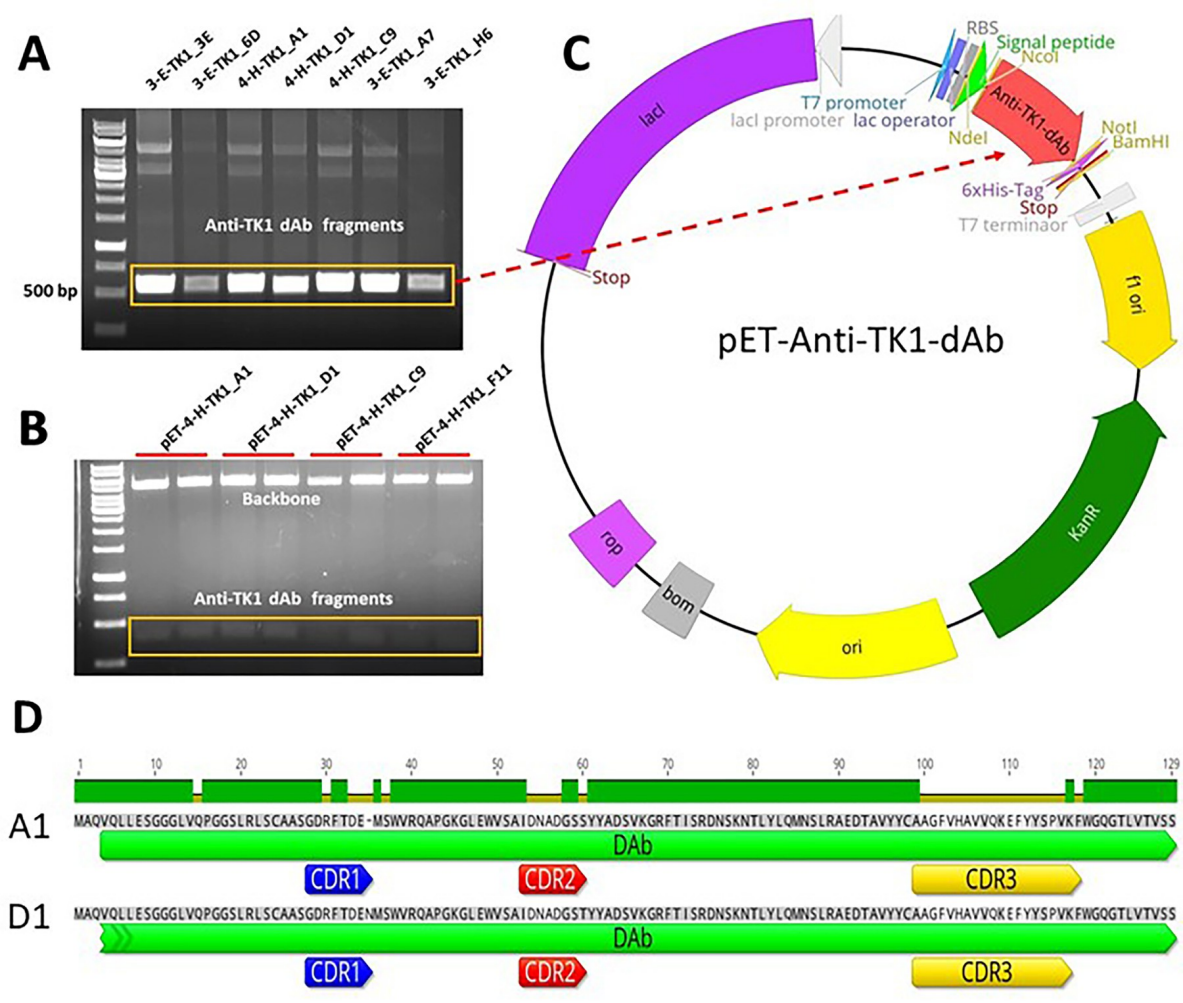
Amplification and ligation of anti-TK1-dAb fragments into the pET-scFv-T expression vector. A) a representative image of a PCR showing amplification of anti-TK1 dAb fragments. B) Restriction enzyme analysis with NcoI and NotI enzymes of pET-anti-TK1-dAb constructs. C) Map of a pET-anti-TK1-dAb construct. Fragments are ligated into the pET-scFv plasmid using NcoI and NotI restriction sites. D) Alignment of the 4-H-TK1_A1 and 4-H-TK1_D1 sdAb sequences shows that the differences of the sdAbs are in their CDRs.

The dAb fragments were amplified by PCR and ligated into the pEt-scFv-T vector successfully as shown in Fig 6. The anti-TK1-sdAb vectors were sequenced, and the amino acid sequences were deduced from their nucleotide sequences (Table 2).

**Table 2:** The deduced amino acid sequences of the best two anti-TK1 sdAb fragments isolated through phage display.

Anti-TK1 dAb	FR1	CDR1	FR2	CDR2
4-H-TK1_A1	MAQVQLLESGGGLVQPGGSLRLSCAA	SGDRFTDEN	MSWVRQAPGKGLEWVSA	IDNADGST
4-HTK1_D1	MAQVQLLESGGGLVEPGGSLRLSCAAS	GDSFTTKN	MAWVRQAPGKGLEWVSA	ISKRSGST
Anti-TK1 dAb	FR3	CDR3	FR4	
4-H-TK1_A1	YYADSVKGRFTISRDNSKNTLYLQMNSLRAEDTAVYYC	AAGFVHAVVQKEFYYSPVKF	WGQGTLVTVSSAAAG	
4-HTK1_D1	YYADSVKGRFTISRDNSKNTLYLQMNSLRAEDTAVYYC	AGLTQRHGHAKLKY	WGQGTLVTVSSAAAG	

Further analysis of the nucleotide sequences of the anti-TK1 dAb fragments with the IgG blast tool from NCBI revealed the specific sites for their respective CDRs. The annotated sequences are shown in Table 2. In addition, comparison of the sequences using genious software confirmed that the annotated CDRs were the regions with most differences while the heavy chain framework regions remained conserved (See Fig 6D). This is consistent with the original description of the library which was built in a human VH framework and introduced diversity in the CDRs.

### Analysis of protein structure of anti-TK1-sdAb fragments and modeling of sdAb-TK1 complexes

**Fig 7:**
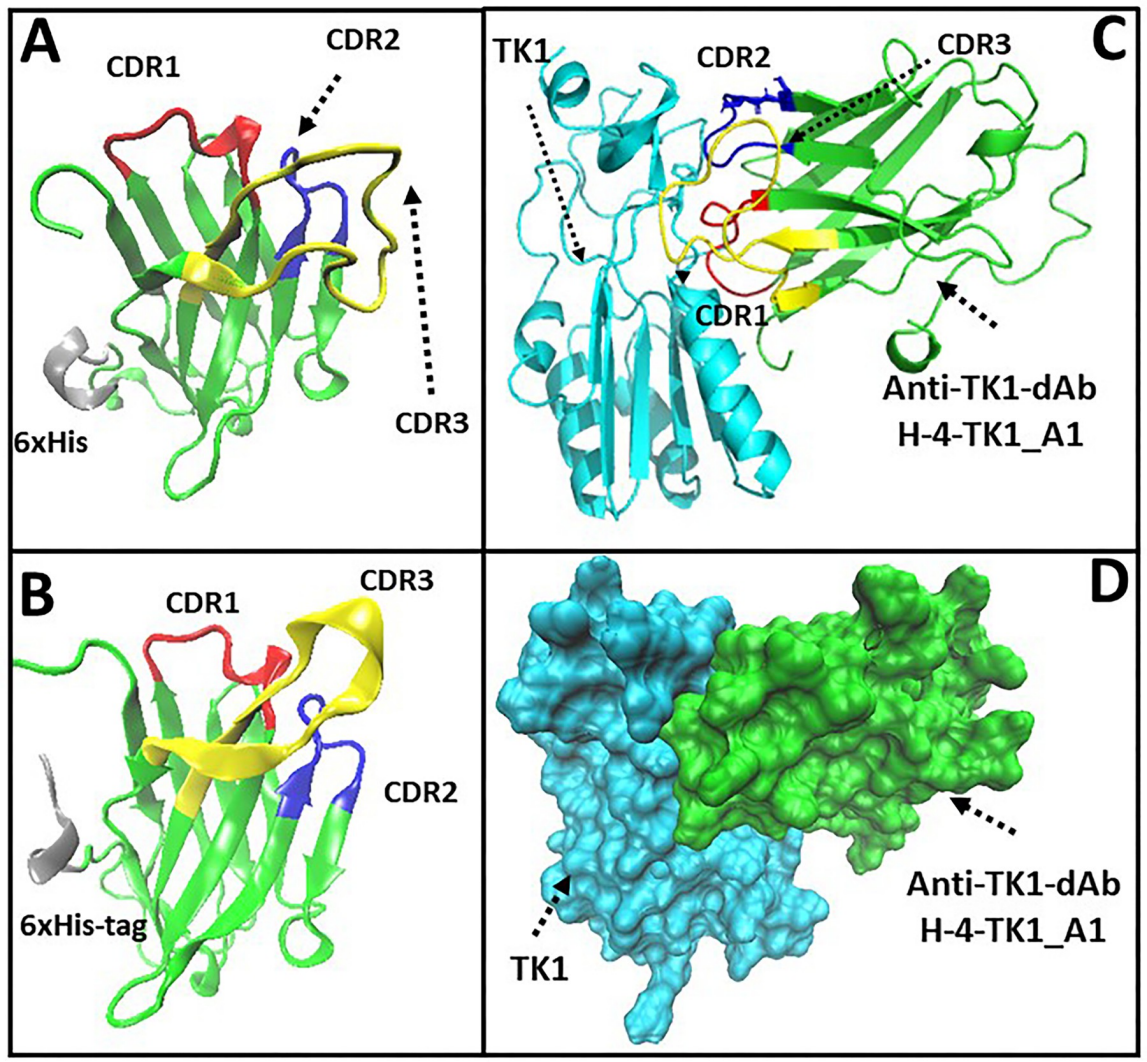
The 3D structure of the two best anti-TK1-sdAb fragments based on their deduced amino acid sequences. The anti-TK1-sdAb fragments were modeled using the GalaxyWeb TBM server. The most stable structures were then visualized using the VMD 1.9.3 software. CDRs were mapped by analyzing the anti-TK1 sdAb amino acid sequences with the IgBlast tool from NCBI. A) H-4-TK1_A1 sdAb. B) H-4-TK1_D1 sdAb. C) High ambiguity driven protein-protein docking analysis using the HADDOCK 2.4 web server. The most stable structures of the H-4-TK1-A1 sdAb fragment and human TK1 protein monomer were analyzed to predict their protein-protein interactions. The analysis shows that in the most stable TK1-TK1sdAb complex, the TK1-sdAb would bind to TK1 through its CDRs. The CDRs would interact with the α1-ribbon towards the N-terminus and two regions close to the β-ribbons towards the c-terminus of the TK1 molecule. D) Docking between the anti-TK1 sdAb H-4-TK1_A1 and the monomer of human TK1 from the 1XBT crystal structure.

After submitting the corresponding amino acid sequences from the anti-TK1 sdAb fragments A1, and D1 into the GalaxyTBM server, 5 model structures were generated for each fragment. The most stable structure from each anti-TK1-sdAb was then visualized using the VMD software. The anti-TK1-sdAb fragments presented a typical structure of a sandwich of two antiparallel β sheets according to previously reported single domain structures [42]. Their CDRs were contained in the loops connecting their antiparallel β sheets with CDR3 displaying a longer loop as expected and in accordance with the sdAb library design (Fig 7A and 7B). Furthermore, analysis of the interaction between the anti-TK1_4-H-A1 sdAb and the 1XBT crystal structure of TK1 using the high ambiguity driven protein-protein docking (HADDOCK) web server revealed the interaction of the anti-TK1 sdAb 4-H-TK1_A1 through its CDRs with TK1 [43]. The CDRs seemed to interact with the α1-ribbon towards the N-terminus and two regions close to the β-ribbons at the c-terminus of the TK1 molecule (Fig 7C and 7D).

### Expression and characterization of purified sdAb fragments

**Fig 8:**
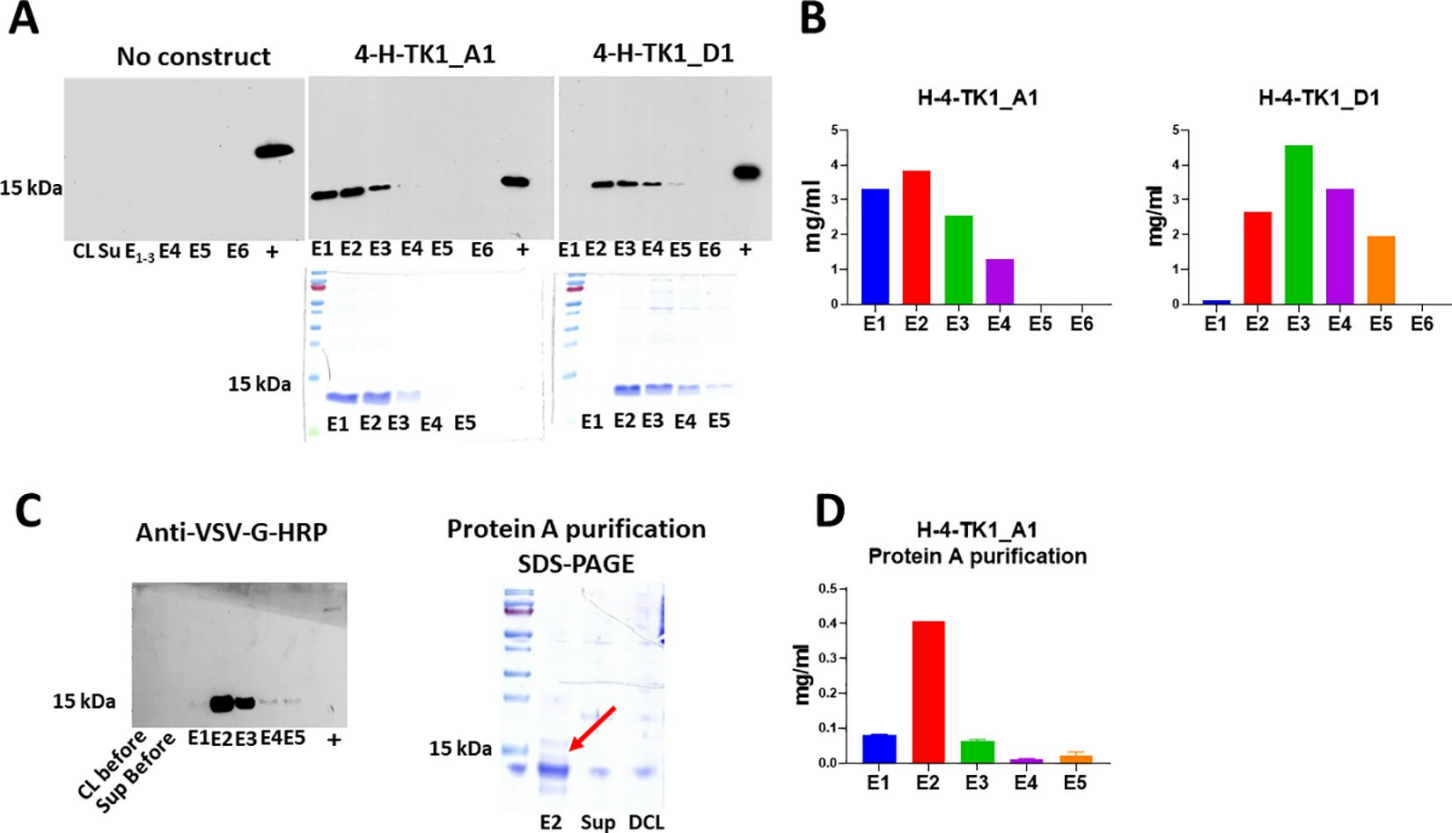
Expression and purification of anti-TK1 sdAbs. A) Detection of anti-TK1 sdAb fragments with anti-His-HRP antibody using Western blot and SDS-PAGE analysis. B) Quantification of the His-tag purified sdAb fragments with BCA assay. The protein yields were between 1–4 mg/ml of purified sdAb. C) Western blot and SDS-PAGE analyses of protein A purified anti-TK1 sdAb H-4-TK1_A1. The antibody fragments can alternatively be purified with protein A purification. D) Quantification of protein A purified anti-TK1 sdAb H-4-TK1_A1.

The anti-TK1 sdAbs H-4-TK1_A1 and H-4-TK1_D1 were successfully expressed using the pET-scFv-T system. The fragments were detected with anti-His-HRP and anti-VSV-G-HRP antibodies in Western blots as could be observed (Fig 8A). Moreover, Coomassie blue staining showed successful purification of a 12–15 kDa band which matched the size of the band shown in Western blots. Purification of sdAb fragments with Ni-NTA bind-His resin yields were between 1 and 4 mg/ml of purified fragment (Fig 8B). Alternatively, the fragments that are VSV-G-tagged were purified using protein A columns (Fig 8C). Although, the yields using protein A purification were lower, about 0.4 mg/ml (Fig 8D).

### Western blot and sdAb ELISA

**Fig 9:**
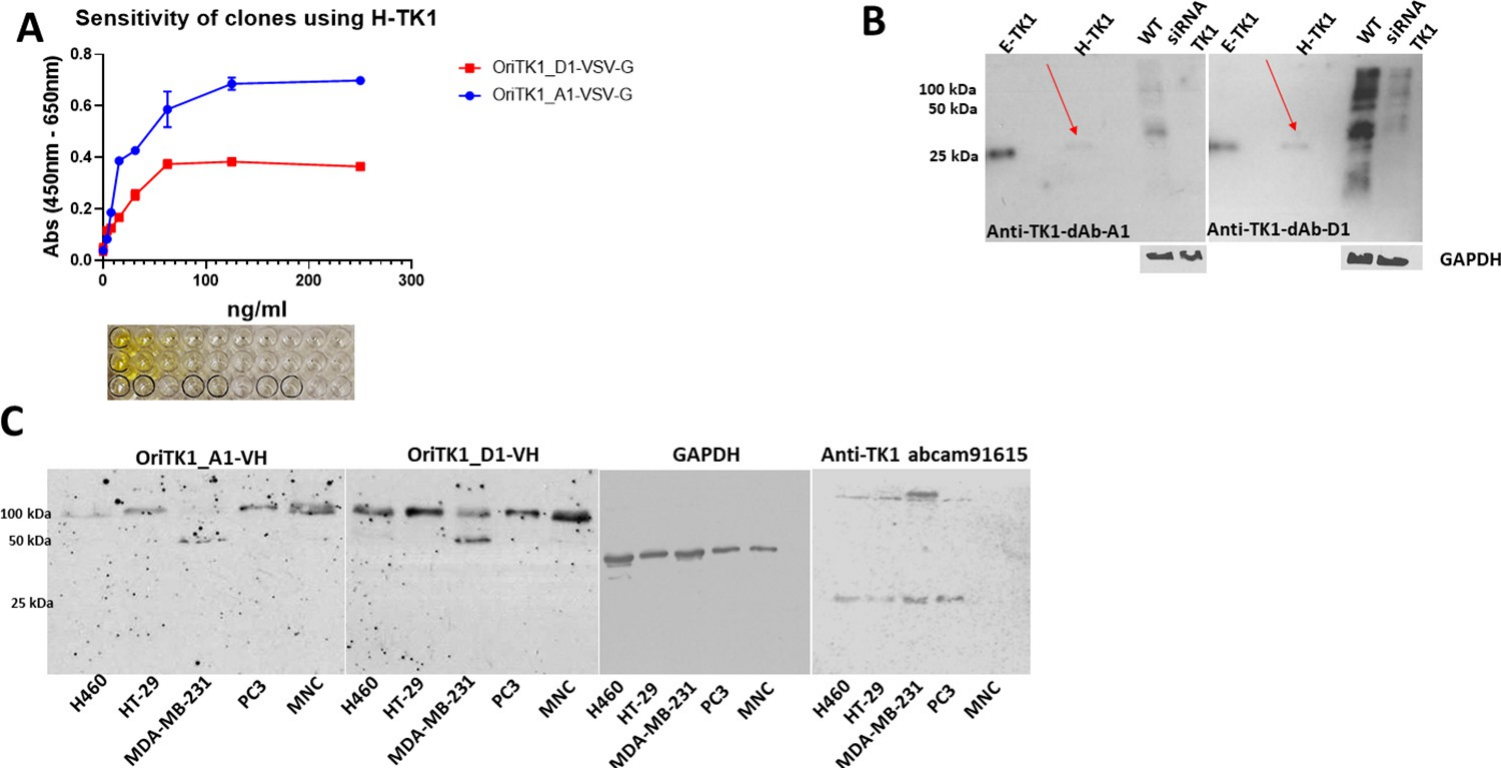
sdAb ELISA and Western blot analyses. A) Purified recombinant anti-TK1 sdAbs kept their binding properties to H-TK1 after being expressed in *E*. *coli* and His-tag purified as observed using sdAb ELISA. B) The anti-TK1 sdAbs showed binding to recombinant human TK1 produced in both *E*. coli (E-TK1) and human cells (H-TK1). The fragments were validated by comparing their binding to TK1 in cell lysates of A549 cells (100 μg) and A549 TK1 knockdown (100 μg). C) Detection of TK1 in cell lysates of 4 different cancer cell lines and normal MNCs (20 μg each). The fragments were able to detect the tetrameric form of TK1, controls included commercial anti-TK1 abcam91651 and GAPDH.

After being expressed as sdAb fragments without a PIII gene fusion, the purified anti-TK1-sdAbs retained binding properties to TK1 similar to those observed when expressed as PIII fusions displayed on filamentous KM13 phage. Thus, indicating that their binding is due to the sdAb sequence and not through non-specific interaction of the coat proteins of the KM13 phage. This was tested using sdAb ELISA. As shown, the anti-TK1 sdAbs bound proportionally to the concentration of TK1 in the wells. Concentrations ranged between 500 ng/ml-3.9 ng/ml of H-TK1. The anti-TK1-sdAbs produced signals significantly higher than the blanks at concentrations of 3.9 ng/ml for H-4-TK1-A1 and for H-4-TK1-D1, with both signals reaching plateau phase after 125 ng/ml (Fig 9A).

The anti-TK1-sdAbs were then tested in Western blot. As can be observed in Fig 9B, the anti-TK1-sdAbs showed binding to recombinant human TK1 produced in *E*. *coli*, and Expi293F cells. Moreover, the anti-TK1 sdAb fragments showed a significantly higher signal in A549 cell lysate compared to A549 TK1 siRNA knockdown. In particular, fragment 4-H-TK1_A1 showed specificity for TK1 (Fig 9B).

Once we confirmed the binding of the fragments to purified recombinant TK1 and validated their specificity using a TK1 siRNA knockdown we proceeded to test their capacity to detect TK1 in cell lysates of different cancer cell lines, including, NCI-H460, HT-29, MDA-MB-231, PC3 and human MNC. It could be observed that the anti-TK1 sdAb OriTK1_A1-VH was able to detect bands corresponding 100 kDa in several cell lysates except in MDA-MB-231 cells where it seems to detect a 50 kDa band instead. Also, the signals for cell lysates from HT-29 and H460 were weaker than the signals coming from other cell lysates. In the case of the anti-TK1 sdAb OriTK1_D1-VH we obtained stronger signals in all cell lysates compared to the signal of OriTK1_A1-VH. OriTK1_D1-VH also detected a 50 kDa band in MDA-MB-231 cells in addition to the 100 kDa band (Fig 9C). These may correspond to the active dimer and tetramer forms of TK1.

### Flow cytometry

**Fig 10:**
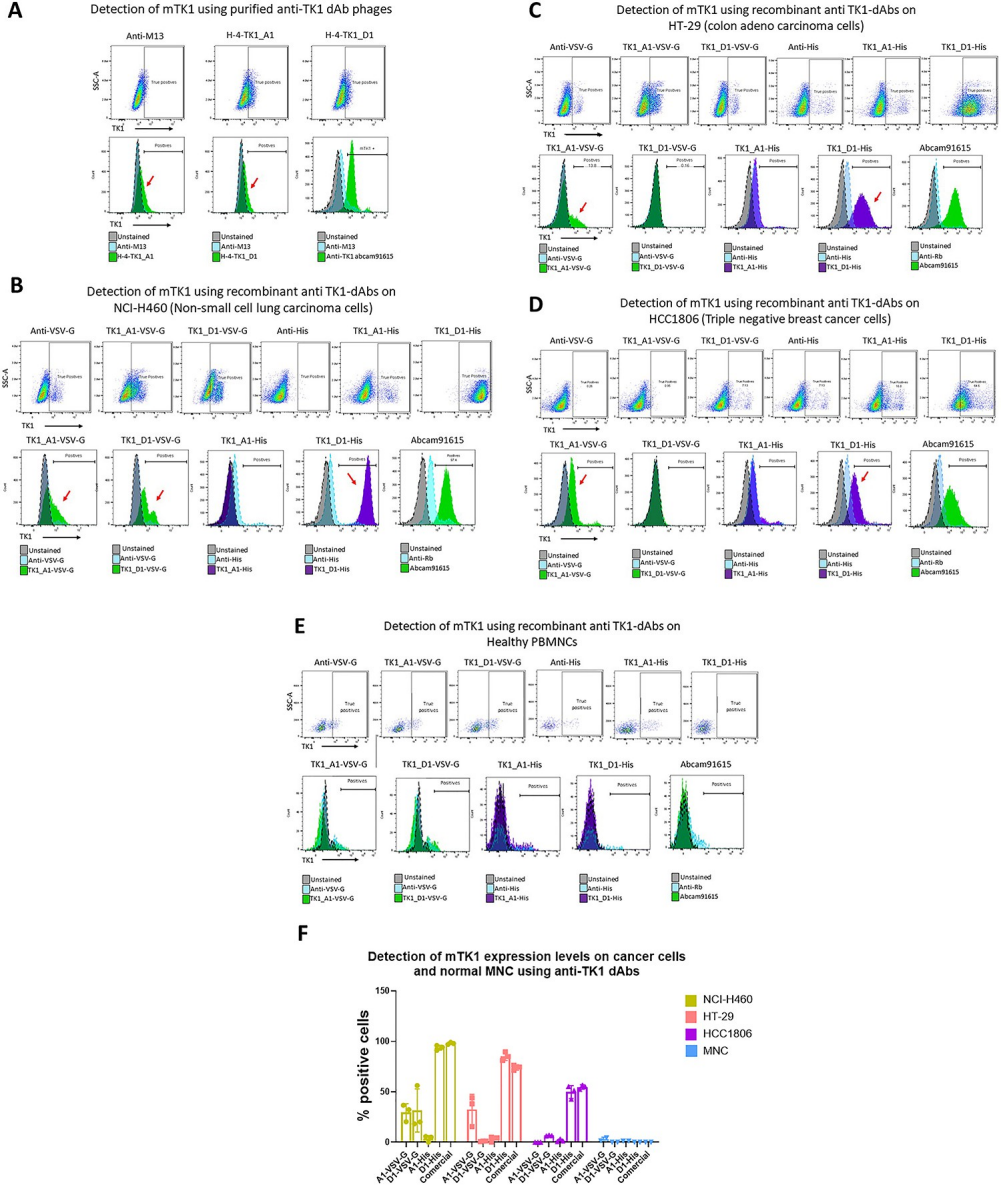
Detection of mTK1 on cancer cells and healthy MNCs using anti-TK1 sdAb fragments. A) NCI-H460 cells were stained with the purified phage-anti-TK1 sdAb fragments, a shift in the population could be observed using both phage-sdAb fragments. B-E) Expressed and purified anti-TK1-sdAb_A1 and D His-tagged and VSV-G tagged were used to stain NCI-H460 (lung), HT-29 (colon), HCC1806 (Triple negative breast cancer) and healthy lymphocytes. The TK1 levels detected using the anti-TK1 sdAb fragments were comparable to the levels detected by commercial anti-TK1 antibody. The highest levels of mTK1 were detected on NCI-H460, followed by HT-29 and then HCC1806. No significant binding was detected on normal MNCs with the anti-TK1 sdAb fragments nor with the commercial anti-TK1 antibody. F) The expression levels of mTK1 on cancer cells and the binding of anti-TK1 sdAb fragments. Only A1-His sdAb showed consistent binding similar to commercial antibody ab91651.

Membrane expression of TK1 on NCI-H460 cells was detected by staining the cells with PEG purified phages from each respective clone. We observed that after subtracting the background binding from the anti-M13-APC antibody there was an increase in binding to the cells stained with the anti-TK1-sdAbs of 20% and 25% for H-4-TK1_A1 and H-4-TK1_D1 sdAbs respectively (Fig 10A). Therefore, the anti TK1 sdAbs showed a capacity to detect membrane expression of TK1 on NCI-H460 cells. NCI-H460 cells were simultaneously screened with the commercial anti-TK1 antibody ab91651.

After confirming the capacity of the TK1-sdAb-phages A1 and D1 to detect mTK1 on cancer cells the fragments were expressed as sdAbs and used to stain different cancer cell lines to confirm their ability to detect mTK1. The sdAbs were expressed as two different versions; His-tagged and VSV-G tagged. Among the 4 sdAbs, TK1-D1-His showed the most consistent binding to mTK1 on NCI-H460(~95%), HT-29(~87%) and HCC1806(~53%) (Fig 10B–10F). These expression levels were comparable to those seen with the commercial TK1 antibody ab91651; 97%, 72% and 55% for NCI-H460, HT-29 and HCC1806 respectively. For NCIH460, which had the highest percentage of cells positive for mTK1, the A1 His-tagged sdAb fragment and both the A1 and D1 VSV-G tagged sdAbs showed binding (10B). D1-VSV-G fragment showed no binding to HT-29 cells or HCC1806 while A1-VSV-G and A1-His showed variable levels of binding to both cell lines. However, not consistently as the D1-His fragment. This may be due to differences in their tags, expression levels in each cell line and possible binding to different epitopes. No significant binding of the anti-TK1 sdAb fragments was found on normal lymphocytes after subtracting non-specific binding of secondary antibody anti-Human IgG FITC. No significant binding of the commercial anti-TK1 antibody was detected either on normal lymphocytes (Fig 10E). It can be appreciated that the cancer cell lines expressed variable levels of mTK1 NCI-H460 being the one with the highest percentage of positive cells followed by HT-29 and HCC1806 (Fig 10F).

### Cloning of the anti-TK1-sdAb fragments into the pFUSE-IgG1e5 vector and expression of recombinant antibody in CHO.K1 cells

**Fig 11:**
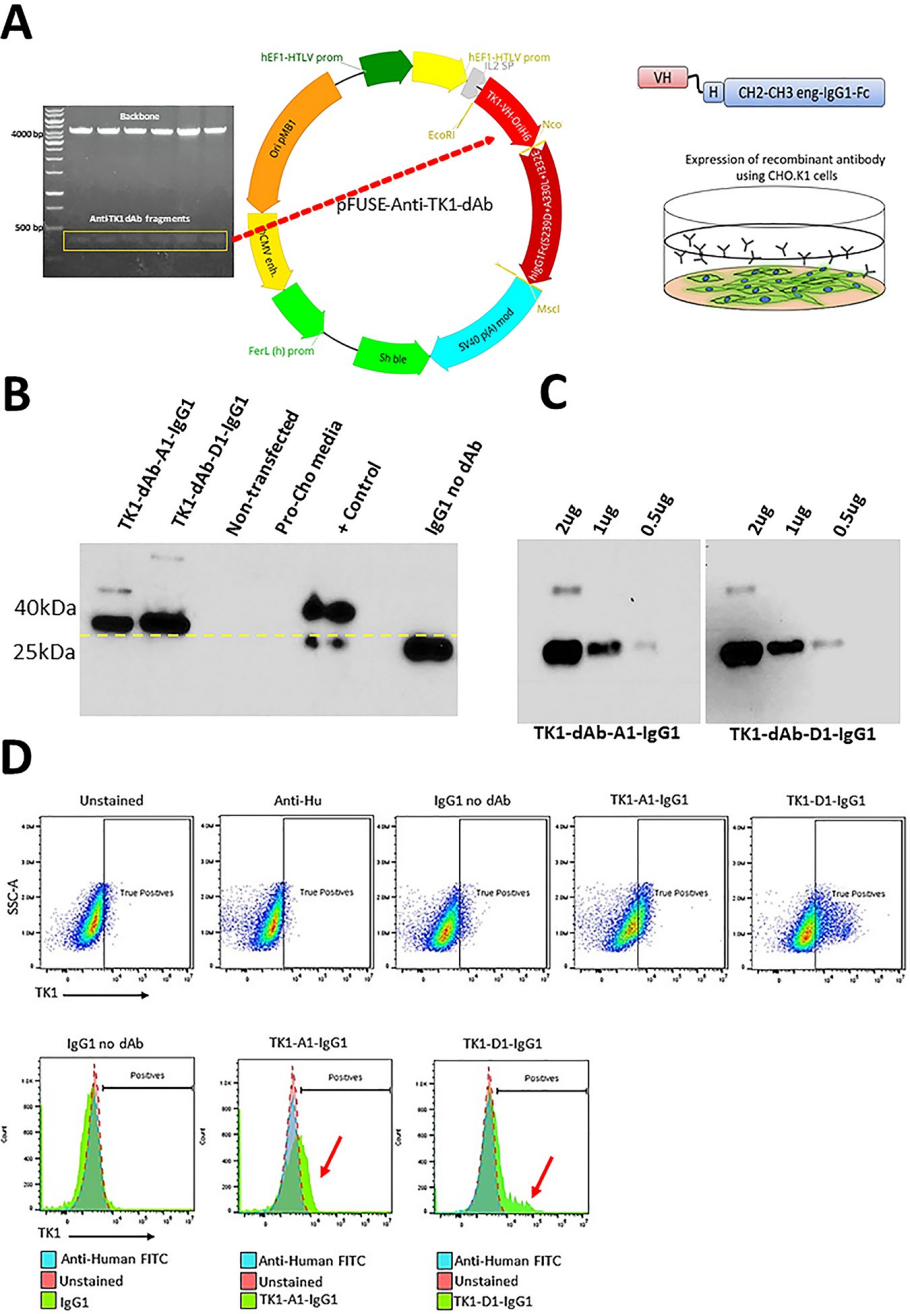
Expression and testing of the Anti-TK1 sDab-IgG1 antibodies. A) Construction of an anti-TK1 sdAb fragment. Restriction analysis of pFUSE-anti-TK1-sdAb plasmids and their respective maps. B) Expression and detection of anti-TK1-sdAb-IgG1 antibodies. As can be observed sdAb-IgG1 fusions produced higher molecular weight bands compared to IgG1-no sdAb constructs. No human IgG was detected in non-transfected CHO.K1 cells. C) After fusing to an Fc the anti-TK1-sdAb antibodies were able to detect mTK1 on NCI-H460 cells. IgG1-no sdAb control did not bind to NCI-H460 cells.

Before cloning the anti-TK1-sdAb sequences into an expression vector, site directed mutagenesis (SDM) was performed to change the amber stop codons present in the sdAb sequences to glutamic-acid. The anti-TK1-sdAb fragments were then successfully ligated into the pFUSE-IgGe5-IL2 expression vector at the NcoI and EcoRI restriction sites. Restriction analysis of these constructs showed the successful insertion of the sdAb genes into the pFUSE-IgGe5-IL2 vector (Fig 11A). After 72 and 96 hours of transfecting CHO.K1 cells with the pFUSE-4-H-TK1_A1 and D1, and a control without sdAb construct, the collected supernatants were purified using a protein A column. The recombinant antibodies were successfully purified from supernatant with a yield of 0.4–0.26 mg/ml. Western blot analysis and SDS page showed for both sdAb-Fc antibodies a ~38–40 kDa band which is the expected size for the monomer of the anti-TK1-sdAb-IgG1 fusion. In the case of the control vector containing an IgG1 sequence without being fused to a sdAb sequence an IgG1 fragment of smaller size (~26 kDa) was produced. The recombinant antibodies were all positive in Western blot to anti-human IgG antibody confirming the presence of the engineered IgG1 heavy chain fused to the sdAbs (Fig 11B). Furthermore, the anti-TK1-sdAbs once fused to IgG1 Fc were able to detect recombinant purified human TK1 (H-TK1). The signal produced in Western blot was proportional to the concentration of antigen and was higher than the signal produced with anti-TK1 sdAbs without FC fusion (Fig 11C). In addition, the recombinant antibodies showed capacity to detect mTK1 on cancer cells in flow cytometry (Fig 11D). A shift in the population of NCI-H460 cells could be seen when they were stained with the TK1-A1-IgG1 and D1 antibodies while no shift was detected using IgG1 fragments without fusion to TK1-sdAbs.

### Anti-TK1-sdAb antibodies elicited in vitro ADCC responses of human MNCs against cancer cells expressing mTK1

**Fig 12:**
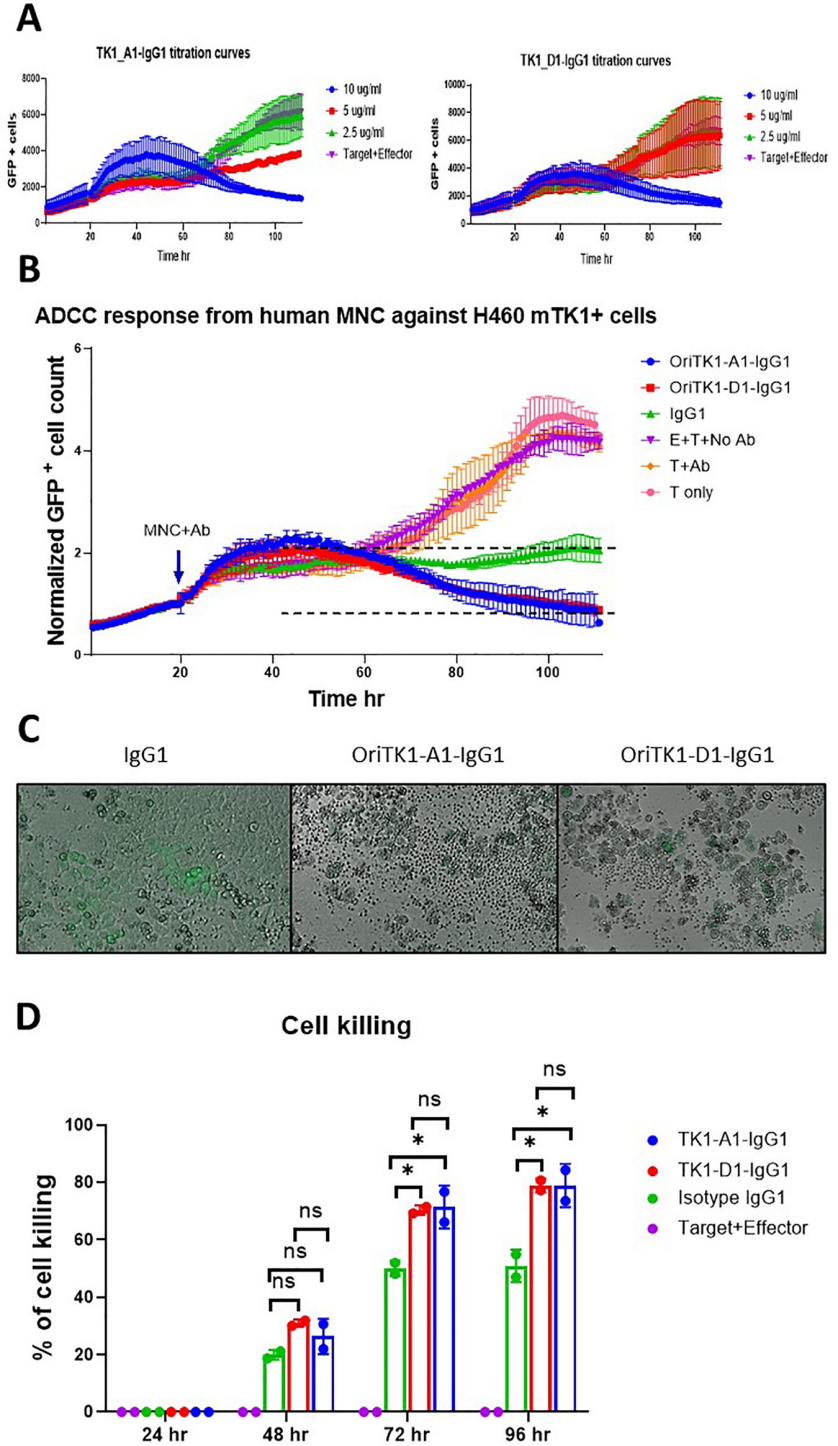
Anti TK1-sdAb-Fc antibodies elicit ADCC responses against NCI-H460 cells expressing mTK1. A) Optimization of several antibody concentrations. The decrease in GFP+ NCI-H460 cells co-cultured with MNC over time is proportional to the concentration of anti-TK1-sdAb-IgG1/ml used. B) A significant ADCC response is elicited by MNCs against NCI-H460 cells when anti-TK1-sdAb-IgG1 antibodies are added compared to controls. After 96 hours the cell killing can visually be appreciated to be more severe in the cells treated with the anti-TK1-sdAb-IgG1 antibodies. C) Percentage of cell killing calculated every 24 hours. The percentage of cell killing was significantly higher after 72 and 96 hours when anti-TK1-sdAbs were added in comparison to IgG1 isotype control and effector only-no sdAb control.

To test the potential use of the anti-TK1-sdAb antibodies for the immunotargeting of cancer cells that express mTK1, we co-cultured tumor cells expressing high levels of TK1 with human MNCs, added anti-TK1-sdAb-Fc antibodies and monitored the ADCC response over time. The NCI-H460 cell line was chosen as this cell was previously shown to express the highest levels of mTK1 on the cell surface in flow cytometry (Fig 11F). An initial test using different concentrations of anti-TK1-sdAbs showed that the cell killing was proportional to the concentration of antibody used (Fig 12A) and it was found that a concentration of 10 μg/ml of the engineered anti-TK1 antibodies was necessary to cause a significant ADCC response. Cells treated with the anti-TK1-sdAb-IgG1_A1 and D1 antibodies at 10 μg/ml and co-cultured with human MNCs had a significant ADCC response against the cancer cells when compared to isotype (*P*<0.0395) and no antibody controls (*P*<0.0038) after 88 hours (Fig 12B). Although the 2-way ANOVA analysis did not show the difference to be significant in the case of the anti-TK1-sdAb-IgG1_D1 antibody, a reduction of more than 50% of target cells was observed compared to isotype controls. Imaging of the cells revealed that after 96 hours there was a difference in the cell health and number of MNCs clustering targets cells. It could be seen that cells treated with anti-TK1-sdAb-IgG1 experienced a more severe ADCC response than those treated with the isotype control (Fig 12B). Statistical analyses at individual time points indicates that the percentage of cell killing is significantly higher at 96 hours when TK1 is targeted using the anti-TK1-sdAb-IgG1_A1 (*P*<0.0267) and D1 (*P*<0.0265) compared to controls and at 72 hours (*P*<0.0207 and *P*<0.0246 respectively) (Fig 12C).

## Discussion

From its early beginnings, studies involving TK1 have been focused mainly on its use as a tumor biomarker [43–45]. However, new evidence has shown that TK1 may have an emerging role as a target for cancer therapy. Recent studies on the suppression of TK1 in cancer cells have shown that the silencing of TK1 decreases the capacity of lung adenocarcinoma, pancreatic and thyroid carcinoma cells to proliferate, migrate or make mesenchymal transitions [23, 24, 46]. Additionally, some TK1 forms seem to be able to associate to the cell membrane of cancer cells, an event that is apparently restricted to malignancy. Thus, it is clear that the development of therapeutics that can specifically target TK1 are necessary to explore the potential of TK1 as a target. Monoclonal antibodies are the fastest growing biopharmaceuticals in immuno-oncology [47]. Previously it has been shown that anti-TK1 monoclonal antibodies could be used for the immunotargeting of TK1 on several cancer types [28]. Although some monoclonal antibodies against TK1 have been developed there are currently no humanized versions of TK1 antibodies suitable for therapy [28, 48]. Moreover, the recent increase in the use of phage display antibody libraries has proven the advantages of using sdAb fragments in the development of therapeutic antibodies for cancer treatment. Single domain antibodies are characterized for their smaller size while keeping all the binding properties of full-length antibodies [49]. In this study anti-TK1 sdAbs were isolated from a sdAb library through phage display. This is the first time that the isolation and characterization of 100% human sdAb fragments specific for TK1 has been reported. This study also provides evidence that single domain antibodies or nanobodies can be used to target mTK1 on cancer cells, an antibody approach that has not been previously used for the targeting of TK1. Furthermore, this study shows evidence that TK1 sdAbs can be incorporated in engineered IgG1 antibody constructs to generate molecules for potential immuno-oncology applications.

It is important to mention that the process through which we selected the antibodies using TK1 produced in bacteria and TK1 produced in human cells was chosen to ensure that we obtained a sufficient number of sdAbs and that the anti-TK1 sdAbs were able to bind to properly folded human TK1. ELISA data in this study has shown that the anti-TK1 sdAbs fit the receptor-ligand model and that the binding of the anti-TK1-sdAb fragments was dependent on the amount of available antigen. This study also showed that the anti-TK1-sdAbs were able to be expressed without the PIII fusion while keeping their binding properties previously shown in phage monoclonal ELISA. Validation with an siRNA TK1 knockdown indicated that the antibody fragments developed were specific for human TK1. Furthermore, the flow cytometry data showed that the nanobodies can potentially be used to target cancer cells expressing TK1, particularly mTK1. This early evidence indicates that anti-TK1-sdAbs could be used for the development of experimental TK1-based therapeutics such as anti-TK1-sdAb fragments that could be conjugated with toxins or gold nanoparticles for anticancer photothermal therapy [50, 51]. Moreover, these anti-TK1-sdAb fragments could also be used for the development of immuno-oncology therapeutics such as engineered antibodies or chimeric antigen receptors [52].

As this study has shown, anti-TK1-sdAb-IgG1 antibodies are capable of targeting mTK1 on cancer cells and elicit an ADCC response by human MNCs against TK1 high-level expressing cancer cells building on previous findings. Unlike previous anti-TK1 antibodies generated through conventional hybridoma technology, these engineered antibodies are completely human, are significantly smaller than full length antibodies and have an engineered IgG1 to enhance the ADCC response. Thus, they can have better tumor penetration than conventional antibodies, and can be used to better engage the immune system with tumor cells. Although it remains unclear why TK1 is localized to the cell surface of multiple cancer cell lines, the flow cytometry data and ADCC results described here suggest that it could be feasible to harness the immune system against tumor cells expressing mTK1. It is not the first time that a protein thought to be limited to the interior of the cell has been reported to be on the cell surface. Examples can be found in the HSP70 family of proteins. Although HSP70 proteins were thought to be limited to function only inside cells, it is well documented that they are secreted and localized on the cell membrane of cancer cells [53, 54]. Recent studies have shown that TK1 is present in exosomes [27]. As with HSP70 proteins a possible explanation could be that it is transitorily localized on the cell membrane as exosomes exit the cell fusing to the cell membrane, and through non-conventional protein-protein interactions. Moreover, it is also important to point out that other nucleotide salvage pathway enzymes have been reported to be localized on the cell membrane e.g., hypoxanthine-phosphoribosyl transferase (HPRT). Targeting of this enzyme with monoclonal antibodies has also been recently reported [55].

## Conclusion

This study reports the isolation and evaluation of human single domain antibodies against TK1 and their binding to the tumor proliferation biomarker TK1 on lung, colon and breast cancer cells. The antibody fragments have potential as diagnostic and therapeutic agents, although additional *in vivo* studies are required to confirm their efficacy. The antibody fragments can be successfully incorporated into IgG1 Fc constructs for the production of completely human engineered antibodies able to elicit significant ADCC responses from human MNCs against cancer cells expressing mTK1. The antibody fragments could potentially be used in other therapies such as chimeric antigen receptors (CAR) for T cells or other recombinant antibody constructs. The use of TK1 as a therapeutic target will enable the testing of experimental TK1-based therapies.

## Supporting information

S1 File Original blot and gel images. (PDF)

S1 Raw images (PDF)
