# Supplementary material for: Selection of human single domain antibodies (sdAb) against thymidine kinase 1 and their incorporation into sdAb-Fc antibody constructs for potential use in cancer therapy
Source: PLoS One. 2022 Mar 3;17(3):e0264822. doi: 10.1371/journal.pone.0264822 (PMC8893706; doi:10.1371/journal.pone.0264822)
Supplement: S1 File — (PDF) [file pone.0264822.s001.pdf]

Original and minimally adjusted blot and gel images. The number of the figure where the original blot/gel or portions of it is shown in the manuscript, is indicated at the beginning of each description.

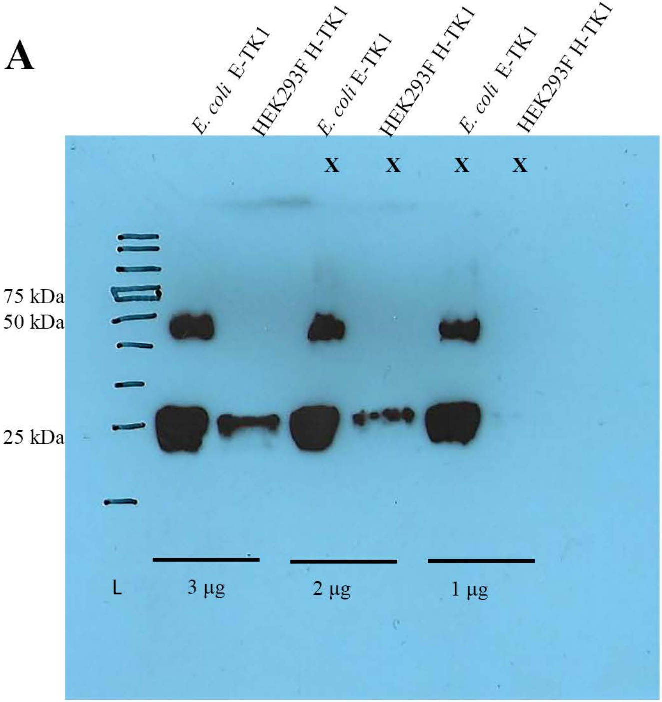

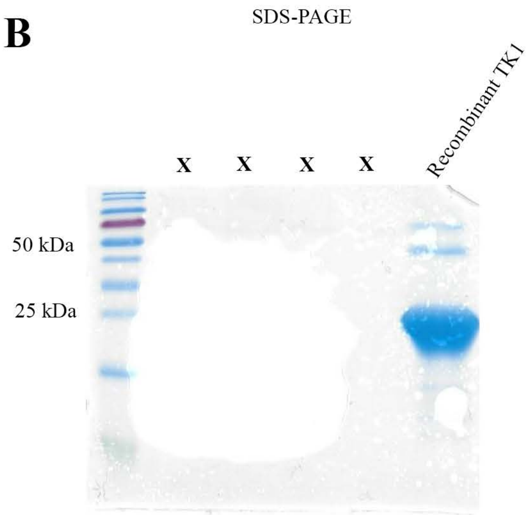

S1 Fig 1 A) Western blot corresponding to purified recombinant TK1 produced in HEK293F and *E. coli*. Various concentrations of both TK1 proteins were tested to optimize Western blot conditions and validate antigen with commercial anti-TK1 antibody ab91651 (Abcam). Only two lanes were shown in Fig 1A . B) Purity of TK1 protein. A purity of 80% was required for screening of sdAb library through phage display. SDS page revealed that the antigen had a purity above 80%. In the case of TK1 produced in HEK293F cells the manufacturer provided purity which was above 80% as well (Origene, TP700037). L= ladder

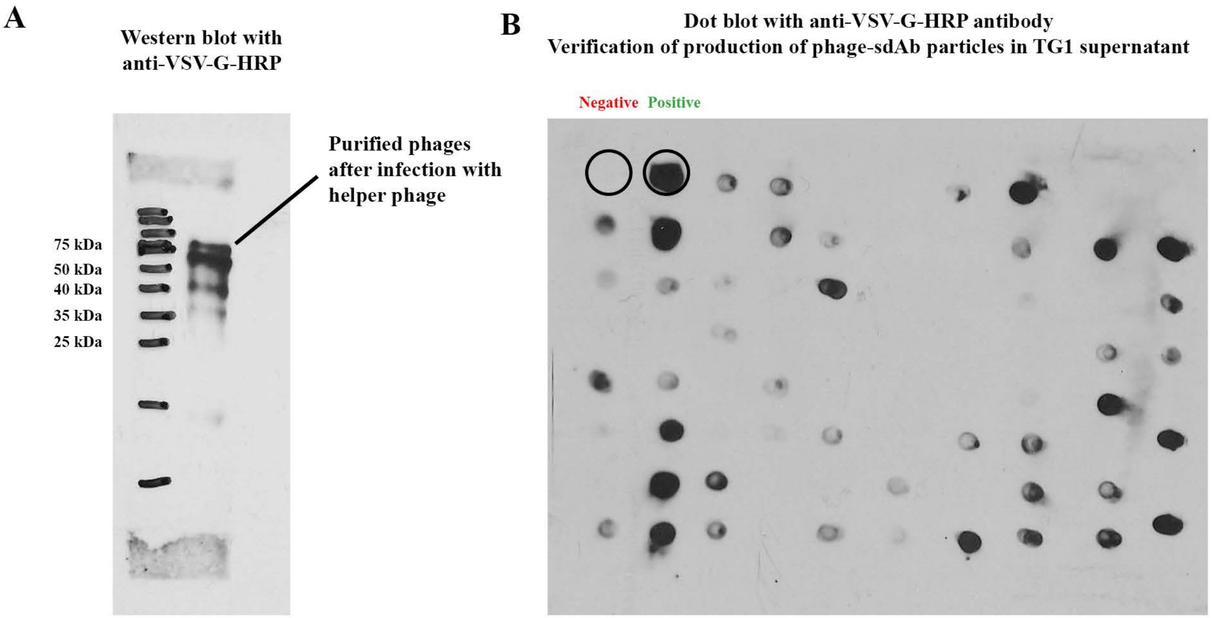

S1 Fig 2C. A) Production of phages displaying sdAbs was verified with anti-VSV-G antibody. TG1 bacteria containing the plasmid phagemid with the sdAb sequences were infected with helper phage to provide all of the packaging proteins. These phages should display sdAbs as a fusion with PIII protein of the phage. Between the sdAb and PIII gene sequence a VSV-G tag has been placed. Only phages displaying an sdAb will also display VSV-G tag. Therefore, the detection of VSV-G in supernatant is an indication of succesful packaging of viral particles and display of sdAb on phages. B) Individual sdAb clones isolated after biopaning were grown in TG1 bacteria and infected with helper phage. After incubating for 24 hours at 25 ºC the supernatants were collected and 2 µl from each clone were analyzed in a dot blot with anti-VSV-G-HRP antibody. A negative control of non-infected TG1 supernatant and a positive control of a 1:1000 dilution of the purified phages from the original dAb library were included. Non infected TG1 did not show any signal from VSV-G antibody. Various clones expressed phages displaying sdAbs as can be seen in the dot blot image.

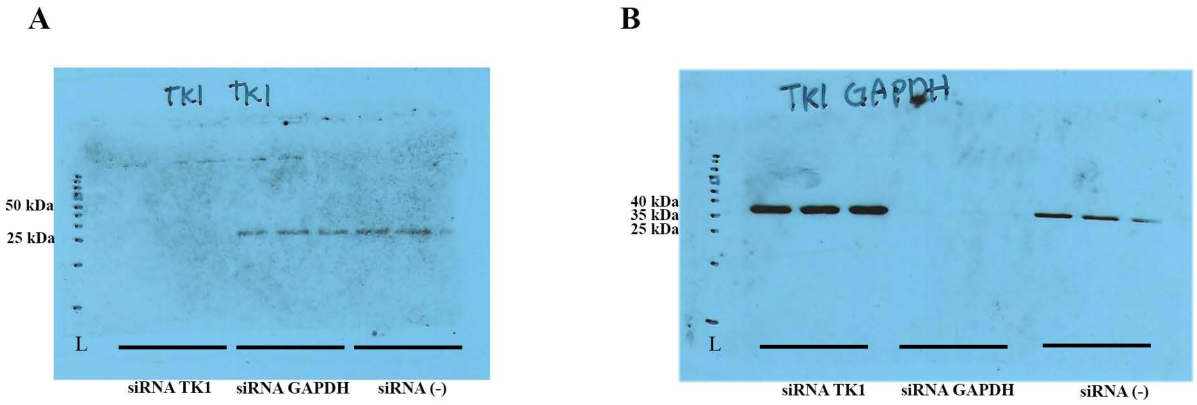

S1 Fig 5C. TK1 knockdown in the non-small cell lung cancer cell line A549 was used to validate the specificity of the anti-TK1 sdAbs 4-H-TK1_A1 and 4-H-TK1_D1. To silence TK1 in A549 cells we used validated TK1 siRNA s14160 Silencer® Select (ThermoFisher Scientific, Waltham, MA). The Silencer™ GAPDH (Cat. No. 4390849) siRNA was used as positive control and the Silencer™ siRNA control No. 1 (Cat. No.4390843) was used as negative control. Cells were transfected using lipofectamine RNAiMAX (Invitrogen, Carlsbad, CA) and 30 pmol of each corresponding siRNA following the manufacturer's protocol. The cells were collected after 72 hours of transfection. Cell lysates were made using RIPA buffer, adding protease inhibitors and 1 mM of phenylmethylsulfonyl fluoride (PMSF). The lysates were cleared by centrifugation and quantified using BCA protein quantification kit. The TK1 knockdown was then validated in Western blot loading 20 µg of protein into each lane for each one of the treatments. Samples were loaded in triplicate and run in SDS-PAGE. The samples were then transferred to a nitrocellulose membrane and the membranes were blocked. After blocking TK1 was detected using the commercial KO validated anti-TK1 antibody ab91651 (abcam, Cambridge, UK), an HRP-conjugated secondary antibody and the enhanced chemiluminescence (ECL) substrate solution (Advansta, San Jose, CA). Images were obtained using light-sensitive film that was exposed to each nitrocellulose membrane and revealed using a developer. A) TK1 knockdown in A549 cells. It can be observed that TK1 was not detected in the first 3 lanes due to treatment with the siRNA TK1. TK1 was detected in the siRNA GAPDH and siRNA (-) controls. B) A positive control was included to evaluate our gene silencing protocol. The anti-GAPDH (14C10) was used to detect GAPDH protein. As it can be observed GAPDH is silenced only in cells treated with the siRNA GAPDH. Silencing of GAPDH or other genes do not silence TK1. As it can be observed in A, TK1 is still present in siRNA GAPDH treated cells. L=ladder

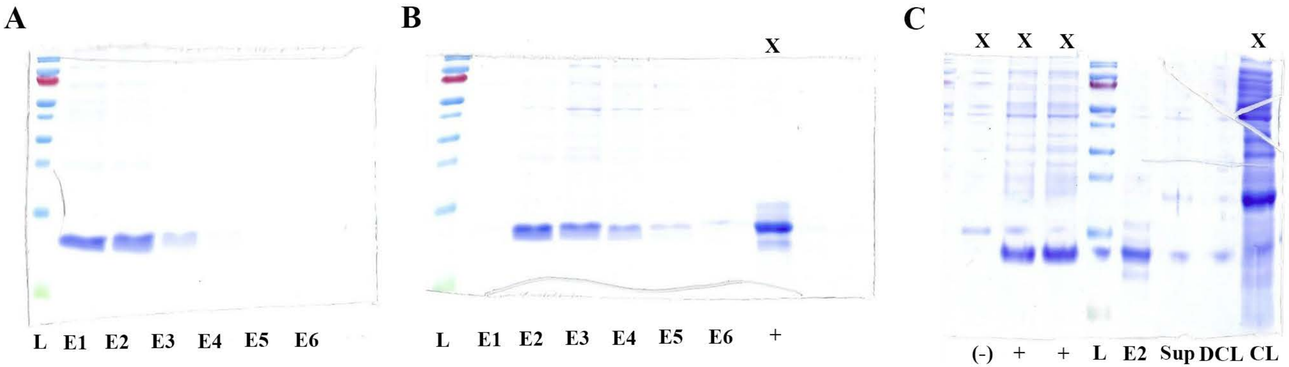

S1 Fig 8A and 8C. SDS-PAGE of purified anti-TK1 sdAb fragments. A) Elution 1-6 of His-tagged purified anti-TK1 4-H-TK1_A1 fragment. B) Elutions 1-6 of His-tagged anti-TK1 4-H-TK1_D1 fragment. Positive sdAb contained in original pET-scFv-T was included. C) SDS-PAGE of Elution 2 from protein A purified anti-TK1 sdAb H-4-TK1_A1. Supernatant, depleted cell lysate and crude cell lysate were included (right side). The left side of the gel are samples non-related to this particular experiment. L= ladder, Sup=Supernatant, DCL=depleted cell lysate, CL=Cell lysate

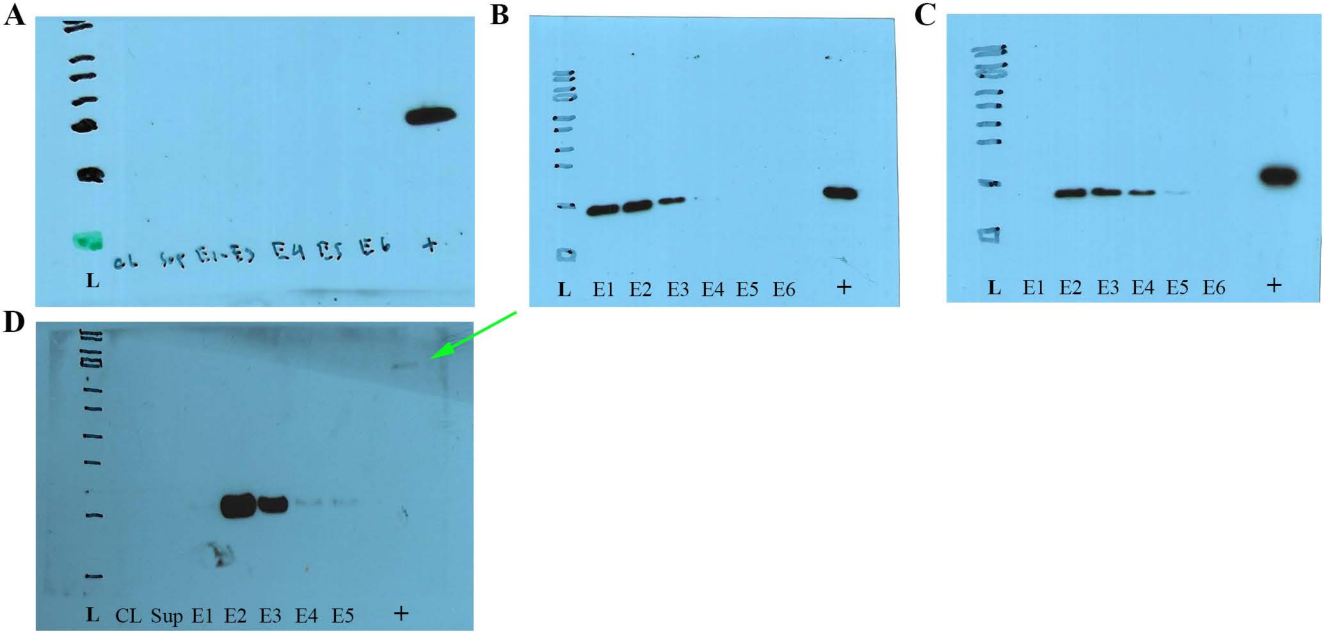

S1 Fig 8A and 8C. Representative Western blot images corresponding to the expression and purification of sdAb OriTK1_A1 and OriTK1_D1. The sdAbs were successfully expressed in bacteria RossetaBlue™(DE3)pLysS cells. Fragments were purified with affinity chromatography using Ni-NTA agarose beads and Protein A resin columns for His-tagged and VSV-G tagged sdAbs respectively. A) Western blot showing the absence of sdAb expression in non-transformed RossetaBlue™(DE3)pLysS cells. This was utilized as our negative control. B and C) Purification of anti-TK1 sdAbs OriTK1A1 (B) and OriTK1D1 (C) with Ni-NTA. Elutions 1-6. An sdAb fragment contained in the pET-scFv plasmid provided by Addgene was used as positive control. D) A representative Western blot image of the purification of VSV-G tagged anti-TK1 sdAb H-4-TK1_A1. The positive controls used were a 1:10,000 dilution of purified phages from the dAb library. L= Ladder, E_n= Elution number, Sup= Supernatant, CL= Cell lysate after running with

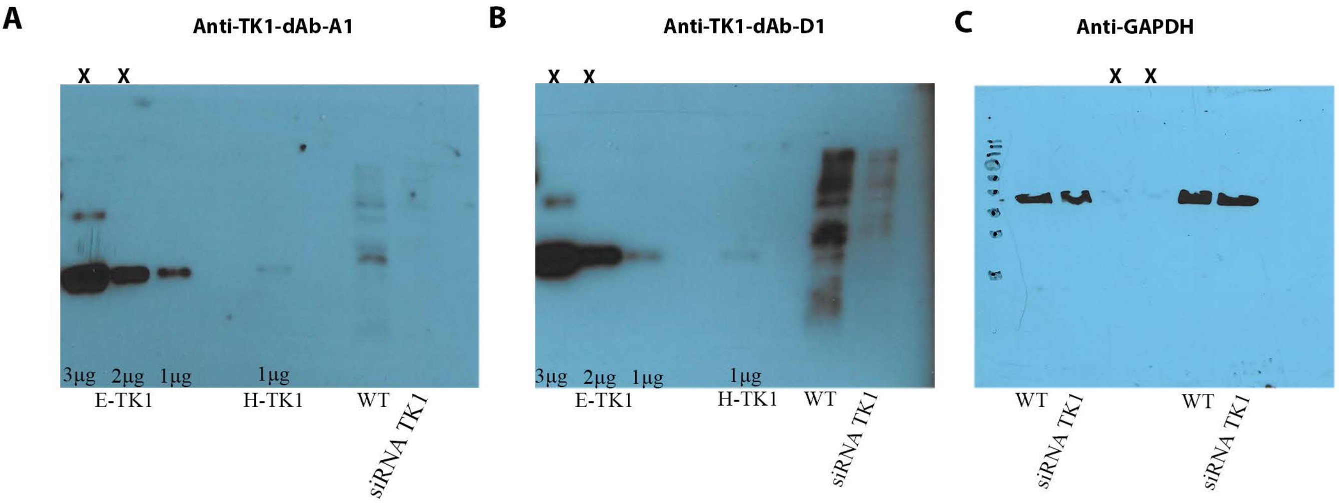

S1 Fig 9B. Representative Western blot images of anti-TK1-sdAb_A1 and anti-TK1-sdAb_D1 binding to purified recombinant human TK1 and validation with siRNA knockout cell line. A) Anti-TK1-sdAb_A1 binds to both E-TK1 and H-TK1. B) Anti-TK1-sdAb_D1 binds to both E-TK1 and H-TK1. The Antibodies specificity to TK1 in cell lysate was tested using WT A549 cell lysate and cell lysate from A549 knockdown. C) The same samples were run simultaneously for GAPDH detection as a control.

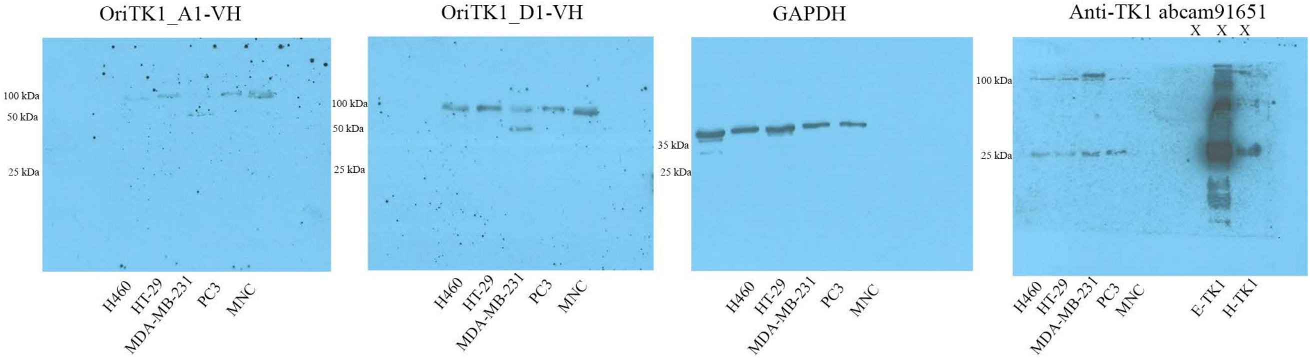

S1 Fig 9C. A representative image of Western blots corresponding to the detection of TK1 using the anti-TK1 sdAb-A1 and sdAb-D1. Five different cell lysates from lung, colon, breast, and prostate cancer cell lines were used. Normal human mononuclear cells were included. The anti-TK1-sdAbs were able to detect a band close to 100 kDa tetramer in cell lysates including normal cell lysate. Only MBA-MD-231 cells produced a lower band of 50 kDa dimer of TK1. The signal was stronger with the anti-TK1-sdAb_D1. The same samples were tested for GAPDH expression and with commercial anti-TK1 antibody.

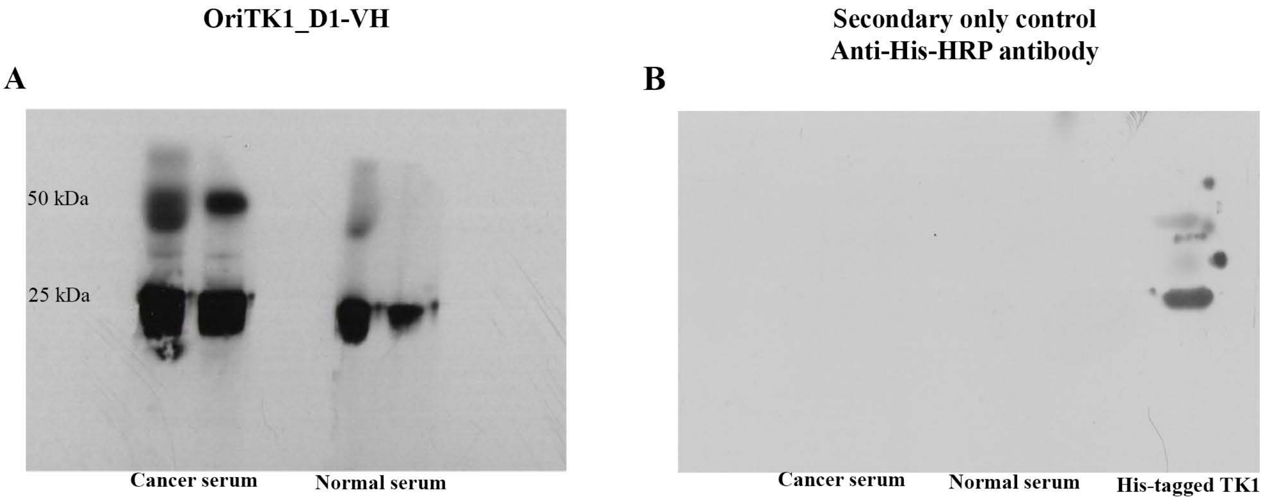

S1 Fig 9D. A representative image corresponding to a Western blot using anti-TK1 sdAb OriTK1_D1-VH for detection of TK1 in human sera from a cancer patient and healthy individual. The sdAb showed binding to TK1 in human serum. A) Bands at 50kDa and 25 kDa corresponding to the dimer and monomer of TK1 can be observed. Serum TK1 from a cancer patient produced a higher signal compared to normal serum. This may be due to higher levels of TK1 in the serum of the cancer patient. B) Secondary only control. No binding of the secondary antibody was detected.

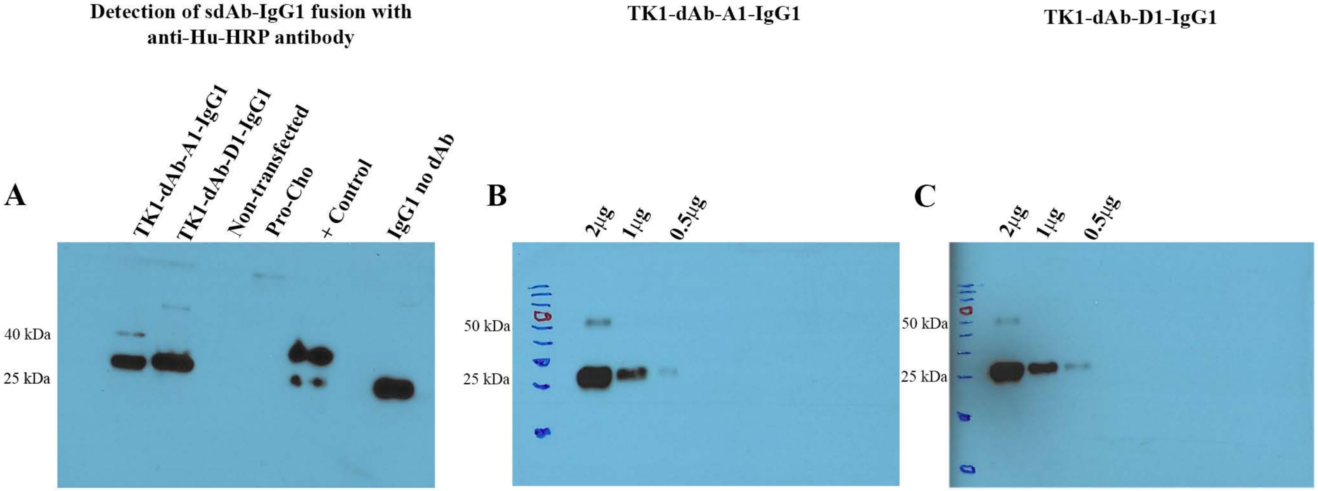

S1 Fig 11B and 11C. A) Expression and purification of anti-TK1-sdAb-IgG1 antibodies. The fusion of a sdAb with human IgG1 produced a slightly higher molecular weight band pf 35-40 kDa. The sdAb-IgG1 antibodies were produced in a CHO-K1 cell line. In the supernatant of non-transduced cells we were not able to detect human IgG. IgG1 no dAb control showed a smaller band than those with the sdAb-IgG1 fusion. B and C) Anti-TK1-sdAb-IgG1 antibodies were able to bind to human TK1 produced in HEK293F cells (H-TK1). The fusion of the IgG1 with sdAb appear to have stabilized the antibody and enhanced its binding, which is reflected in a stronger signal compared to what was observed with the sdAbs without IgG1 fusion.
